# Associations of six adiposity-related markers with incidence and mortality from 24 cancers—findings from the UK Biobank prospective cohort study

**DOI:** 10.1186/s12916-020-01848-8

**Published:** 2021-01-11

**Authors:** Solange Parra-Soto, Emma S. Cowley, Leandro F. M. Rezende, Catterina Ferreccio, John C. Mathers, Jill P. Pell, Frederick K. Ho, Carlos Celis-Morales

**Affiliations:** 1 Institute of Health and Wellbeing, University of Glasgow, Glasgow, G12 8RZ UK; 2 Institute of Cardiovascular and Medical Sciences, University of Glasgow, Glasgow, G12 8TA UK; 3 Departamento de Medicina Preventiva, Universidade Federal de São Paulo, Escola Paulista de Medicina, Sao Paulo, Brazil; 4 Advanced Center for Chronic Diseases, Universidad de Chile and Pontificia Universidad Católica de Chile, Santiago, Chile; 5 Facultad de Medicina, Pontificia Universidad Católica de Chile, Santiago, Chile; 6 Human Nutrition Research Centre, Population Health Sciences Institute, Newcastle University, Newcastle upon Tyne, NE2 4HH UK; 7 Centro de Investigación en Fisiología del Ejercicio (CIFE), Universidad Mayor, 7510041 Santiago, Chile; 8 Laboratorio de Rendimiento Humano, Grupo de Estudio en Educación, Actividad Física y Salud (GEEAFyS), Universidad Católica del Maule, 3480112 Talca, Chile

**Keywords:** Obesity, Body mass index, Waist circumference, Body fat, Cancer, UK Biobank

## Abstract

**Background:**

Adiposity is a strong risk factor for cancer incidence and mortality. However, most of the evidence available has focused on body mass index (BMI) as a marker of adiposity. There is limited evidence on relationships of cancer with other adiposity markers, and if these associations are linear or not. The aim of this study was to investigate the associations of six adiposity markers with incidence and mortality from 24 cancers by accounting for potential non-linear associations.

**Methods:**

A total of 437,393 participants (53.8% women; mean age 56.3 years) from the UK Biobank prospective cohort study were included in this study. The median follow-up was 8.8 years (interquartile range 7.9 to 9.6) for mortality and 9.3 years (IQR 8.6 to 9.9) for cancer incidence. Adiposity-related exposures were BMI, body fat percentage, waist-hip ratio, waist-height ratio, and waist and hip circumference. Incidence and mortality of 24 cancers sites were the outcomes. Cox proportional hazard models were used with each of the exposure variables fitted separately on penalised cubic splines.

**Results:**

During follow-up, 47,882 individuals developed cancer and 11,265 died due to cancer during the follow-up period. All adiposity markers had similar associations with overall cancer incidence. BMI was associated with a higher incidence of 10 cancers (stomach cardia (hazard ratio per 1 SD increment 1.35, (95% CI 1.23; 1.47)), gallbladder (1.33 (1.12; 1.58)), liver (1.27 (1.19; 1.36)), kidney (1.26 (1.20; 1.33)), pancreas (1.12 (1.06; 1.19)), bladder (1.09 (1.04; 1.14)), colorectal (1.10 (1.06; 1.13)), endometrial (1.73 (1.65; 1.82)), uterine (1.68 (1.60; 1.75)), and breast cancer (1.08 (1.05; 1.11))) and overall cancer (1.03 (1.02; 1.04)). All these associations were linear except for breast cancer in postmenopausal women. Similar results were observed when other markers of central and overall adiposity were used. For mortality, nine cancer sites were linearly associated with BMI and eight with waist circumference and body fat percentage.

**Conclusion:**

Adiposity, regardless of the marker used, was associated with an increased risk in 10 cancer sites.

**Supplementary information:**

The online version contains supplementary material available at 10.1186/s12916-020-01848-8.

## Background

Currently, 67% of men and 62% of women are overweight or obese in the UK. Obesity has strong association with increased incidence of, and premature mortality from, some types of cancer [1, 2]. A recent report by the World Cancer Research Fund (WCRF) summarises the evidence showing that high BMI is associated with higher risk of 12 cancers, including colorectal, breast in postmenopausal women, oesophageal, pancreatic, liver, kidney, oral, pharynx and larynx, stomach cardia, gallbladder, ovarian, (advanced) prostate, and womb cancers [3]. However, the WCRF report also highlighted the lack of evidence regarding the association of cancer with other markers of adiposity (i.e. central adiposity and body fat).

Although previous studies have reported the association of several cancer sites with different markers of adiposity [2, 4, 5], most of these studies have been conducted in Asian populations [6, 7], Lee et al. reported the associations of 18 cancers with waist circumference (WC) in 22.9 million Korean adults [7]. Similarly, Wang et al. reported the associations of four markers of adiposity including BMI, WC, waist-to-hip ratio (WHR), and body fat percentage (BF%) with 15 cancers in the China Kadoorie Biobank [6]. Evidence derived from white or British populations has focused mainly on a small number of cancer sites (i.e. breast, colon, endometrium, and prostate) [8–11], or has been restricted to BMI as a marker of adiposity [2, 4, 5]. In 2014, Bhaskaran et al. [2] reported that BMI was associated with 17 cancers in 5.2 million British adults. This study also highlighted the need for further evidence for other adiposity markers since measures of body fat distribution, such as central obesity and body fat might be stronger determinants of specific cancer sites than BMI [12], as observed for other health outcomes such as cardiovascular diseases [13]. Moreover, most of the evidence available to date have assumed a linear association between markers of adiposity and cancer risk from most common sites (colorectal, breast cancer, liver, kidney, and gallbladder) [4, 12], with a limited number of studies investigating non-linear association [2, 14, 15]. To address these limitations, we used data from the UK Biobank cohort, a large prospective cohort study, to investigate the associations of six adiposity markers with incidence and mortality from 24 cancers by accounting for potential non-linear associations.

## Methods

### Study design

UK Biobank recruited more than 500,000 participants (aged 37–73 years, 56.3% were women) between 2006 and 2010 [16]. Participants attended one of 22 assessment centres across England, Scotland, and Wales, where they completed a self-administered, touch-screen questionnaire and face-to-face interviews [17, 18]. After excluding participants with a prevalent cancer diagnosis at baseline (*n* = 41,460), those with missing data for exposures and covariates (*n* = 21,064), and participants who were classified as underweight (*n* = 2629), 437,393 participants were finally included in the study. The outcomes defined for this study were incidence and mortality of overall cancer and 24 specific cancers. Of the 24 cancers, 17 were relevant to both men and women, two were specific to men (testicular and prostate cancer), and five were specific to women (breast, endometrium, uterine, cervix and ovary). The exposures were six adiposity-related markers, including BMI, WC, WHR, waist-to-height ratio (WHtR), hip circumference (HC), and BF%. The covariates were sociodemographic factors (age, ethnicity, education, and Townsend deprivation), smoking status, dietary intake (red meat, processed meat, fruit and vegetables, oily fish, and alcohol), physical activity, and sedentary behaviour. Additional cancer-specific covariates were added for women-related cancer (hormonal replacement, ages at first live birth, last live birth, and at menarche). Additionally, sun exposition was added as a covariate for melanoma cancer, and for lung, oesophageal, and oral cancer, we restricted the analysis to never smoker only. Association between adiposity markers and cancer mortality is likely the combined effect of adiposity’s association with incident cancer, and adiposity’s association with cancer fatality among cancer patients.

### Procedures

Date of death was obtained from death certificates held within the National Health Service Information Centre (England and Wales) and the National Health Service Central Register Scotland (Scotland). Date and cause of hospital admissions were obtained through record linkage to Health Episode Statistics (England and Wales) and Scottish Morbidity Records (Scotland). Detailed information about the linkage procedures can be found at http://content.digital.nhs.uk/services. At the time of analysis, mortality data were available up to 01 June 2020. Mortality analysis was therefore censored at this date or date of death, whichever occurred earlier. Hospital admission data were available until 31 March 2017 for Scotland and Wales and until 01 June 2020 for England, resulting in analyses of incident outcomes being censored at this date or the date of relevant hospitalisation or death, whichever occurred earlier. We defined incident cancer as fatal or nonfatal events. The International Classification of Diseases, 10th revision (ICD-10), was used to define the following 27 cancers: overall cancer (C00–C97, D37, D48), oral (lip, pharynx and larynx) (C00–C14), oesophagus (C15) upper oesophagus (C15.0, 15.1, 15.3, and 15.4), stomach (C16) stomach cardia (C16.0), stomach non cardia (C16.1–16.6), colorectal (C18, C19, and C20), colon proximal (C 18.0–18.5), colon distal (C18.6, C18.7), colon (C18.0-C18.9), rectum (C19–C20), liver (C22), gallbladder (C23), pancreas (C25), lung (C34), malignant melanoma (C43), breast (C50), uterine (C54–C55), cervix (C53), endometrium (C54), ovary (C56), prostate (C61), testis (C62), kidney (C64-C65), bladder (C67), brain (C71), thyroid (C73), lymphatic and haematopoietic tissue (C81–C96), non-Hodgkin lymphoma (C82–C85), multiple myeloma (C90), and leukaemia (C91–C95).

The exposures were six adiposity-related markers (BMI, WC, WHR, WHtR, HC, and BF%) measured by trained staff using standardised protocols across the assessment centres at baseline. Height was measured to the nearest centimetre, using a Seca 202 stadiometer, and body weight to the nearest 0.1 kg, using a Tanita BC-418 body composition analyser. BMI was calculated as weight (kg) divided by height (m) squared and classified into the following categories: underweight (< 18.5 kg/m^2^), normal weight (18.5 to < 25 kg/m^2^), overweight (25 to < 30 kg/m^2^), and obese (> 30 kg/m^2^) [19]. 

BF% was measured using the Tanita BC-418 MA body composition analyser (fat mass divided by the total body mass).

The natural indent was used to measure WC (the umbilicus was used if the natural indent could not be observed) and used to determine central obesity (WC ≥ 88 cm for women and WC ≥ 102 cm for men). HC was recorded at the widest part of the hips. WHR and WHtR are the ratios of the waist-to-hip circumference and waist circumference to height, respectively.

Age, sex, ethnicity, smoking status, diet (portions of fruits and vegetables, red and processed meat, and oily fish) and alcohol intake (daily, 2–4 times a week, once or twice a week, 1–3 time a month, special occasions and never), sun exposition (do not go out in the sunshine, rarely, sometimes, most of the time, always), and female-specific factors were self-reported at the baseline assessment by touch-screen questionnaire. Townsend area deprivation index was derived from the postcode of residence using aggregated data on unemployment, car and homeownership, and household overcrowding [20]. Educational qualification was self-reported. Physical activity level over a typical week was self-reported using the International Physical Activity Questionnaire and reported as metabolic equivalent of task (MET) per week [21]. Time spent on discretionary sedentary behaviours was derived from the questionnaire and included time spent in front of a TV or computer or driving during leisure time. Further details of these measurements can be found in the UK Biobank online protocol (http://www.ukbiobank.ac.uk).

### Statistical analyses

Cox proportional hazard models were used to estimate hazard ratios (HR) and 95% confidence intervals for each adiposity marker (BMI, WC, BF%, WHR, WHtR, and HC) separately with incidence and mortality for 24 cancers and all-cause cancer. Duration of follow-up was used as the timeline variable. The exposure variables were fitted separately on penalised cubic splines to investigate non-linear associations between each adiposity exposure and the outcomes. Penalised spline is a variation of basis spline [22]. Non-linearity was tested by likelihood ratio tests. To compare the associations between cancer across different adiposity markers, all adiposity exposures were standardised by sex and HR were expressed per 1-standard deviation increment (1-SD was equivalent to BMI units of 4.2 and 5.1 kg/m^2^, WC 11.3 and 12.5 cm, WHR 0.07 and 0.07, WHtR 6.5 and 7.9, HC 7.6 and 10.4 cm, BF% 5.8 and 6.9%, and BFI 2.6 and 3.8 kg/m^2^ for men and women, respectively). Participants with prevalent cancer at the baseline assessment were excluded from the study (*n* = 41,406). Underweight participants were also excluded from the study (*n* = 2629). In addition, a landmark analysis was performed to reduce the potential for reverse causality, with follow-up commencing 2 years after recruitment. The association between adiposity and oesophageal, oral, and lung cancer was restricted to participants who reported being never smokers, to avoid reverse causation bias. For breast cancer, all analyses were stratified by menopausal status. Additional sensitivity analyses were performed including underweight people and adding height as a covariate.

Population attributable fractions (PAFs), assuming causality, were calculated based on the BMI distribution of Health Surveys of England, Scotland, and Wales in 2018 [23–25] and the HRs derived from this study using the standard formula with 95% confidence interval (CI) and *P* values estimated using bootstrapping (formula shown in Additional file 1: Figure S1) [26].

To compare cancer risk discrimination between BMI and the remaining five adiposity markers, we calculated Harrell’s C-index (the probability of concordance between observed and predicted responses) for a model that included the adiposity marker and covariates (age, ethnicity, deprivation, education, smoking, alcohol consumption, intakes of fruit and vegetables, red and processed meat, oily fish, physical activity, and sedentary behaviours). The model with BMI was defined as baseline model. The C-indices of the baseline model and the C-index difference between other adiposity model and the baseline model were reported. The variance of the C-indices was calculated using the formula as described previously [27]. These were then used to calculate confidence intervals and *P* values using normal approximation.

Competing risk due to non-cancer mortality was handled using a cause-specific model [28]. Participants who died due to non-cancer causes were marked as censored at their date of death. This approach was used instead of the sub distribution proportional hazards model because there is no evidence that the competing events influence the risk of cancer events, and because the current study aims to investigate associations rather than absolute risk.

Finally, because of potentially inflated type I errors due to multiple tests, all analyses were corrected for multiple testing using Holm’s method [29], which performed similarly to Bonferroni’s method while retaining higher statistical power [30]. The multiple testing corrected *P* value are denoted as *P*_adj_ for *P* value for testing overall significance against no association, and P_nonlinear_ for *P* value testing non-linearity.

All analyses were adjusted for age, sex, ethnicity, deprivation, education, smoking, alcohol consumption, intakes of fruit and vegetables, red and processed meat, oily fish, physical activity, and sedentary behaviours. Additionally, women-related cancer was further adjusted for hormonal replacement, age at menarche, and age at first and last live birth. Prostate cancer was additionally adjusted for family history of prostate cancer, and melanoma was further adjusted for sun exposure. All analyses were performed using R Statistical Software, version 3.6.2, with the package survival and pifpaf.

## Results

This study included 437,393 participants who were followed up for 8.8 years (interquartile range (IQR) 7.9 to 9.6) for cancer incidence and 9.3 (IQR 8.6 to 9.9) for cancer mortality, after excluding the 2-year landmark analysis. Over this period, 47,882 incident cancer cases and 11,265 cancer deaths occurred (Additional file 1: Table S1 and S2). The characteristics of participants stratified by BMI categories are shown in Table 1. In summary, 53.8% of the study population were women, 94.6% were of white European background. The mean population age was 56.3 years, 55.3% of subjects had never smoked, and 10.4% were current smokers.

**Table 1:** Cohort baseline characteristics

	Normal weight	Overweight	Obese	Overall
*n*	143,460 (32.8%)	187,563 (42.9%)	106,370 (24.3%)	437,393
Age, mean (SD)	55.4 (8.22)	56.7 (8.07)	56.6 (7.90)	56.3 (8.10)
Sex				
Females	92,922 (64.8%)	87,097 (46.4%)	55,246 (51.9%)	235,265 (53.8%)
Males	50,538 (35.2%)	100,466 (53.6%)	51,124 (48.1%)	202,128 (46.2%)
Townsend deprivation index				
Lower deprivation	51,511 (35.9%)	65,530 (34.9%)	30,740 (28.9%)	147,781 (33.8%)
Middle deprivation	48,183 (33.6%)	63,918 (34.1%)	34,366 (32.3%)	146,467 (33.5%)
Higher deprivation	43,766 (30.5%)	58,115 (31.0%)	41,264 (38.8%)	143,145 (32.7%)
Education				
College or University degree	64,263 (44.8%)	69,351 (37.0%)	32,442 (30.5%)	166,056 (38.0%)
A levels/AS levels or equivalent	17,455 (12.2%)	20,738 (11.1%)	11,116 (10.5%)	49,309 (11.3%)
O levels/GCSEs or equivalent	29,336 (20.4%)	40,223 (21.4%)	23,510 (22.1%)	93,069 (21.3%)
SEs or equivalent/NVQ or HND or HNC or equivalent	13,885 (9.7%)	23,548 (12.6%)	15,352 (14.4%)	52,785 (12.1%)
Missing	18,521 (12.9%)	33,703 (18.0%)	23,950 (22.5%)	76,174 (17.4%)
Ethnicity				
White	136,331 (95.0%)	177,574 (94.7%)	99,866 (93.9%)	413,771 (94.6%)
Mixed	2101 (1.5%)	2703 (1.4%)	1741 (1.6%)	6545 (1.5%)
South Asian	2830 (2.0%)	3965 (2.1%)	1869 (1.8%)	8664 (2.0%)
Black	1327 (0.9%)	2905 (1.5%)	2813 (2.6%)	7045 (1.6%)
Chinese	871 (0.6%)	416 (0.2%)	81 (0.1%)	1368 (0.3%)
Height (m), mean (SD)	1.68 (0.08)	1.69 (0.09)	1.68 (0.09)	1.69 (0.09)
Weight (kg), mean (SD)	64.7 (8.47)	78.6 (9.63)	95.9 (14.3)	78.2 (15.8)
Waist circumference (cm), mean (SD)	78.6 (8.10)	91.0 (8.36)	105 (11.0)	90.3 (13.3)
Body mass index (kg/m^2^), mean (SD)	22.9 (1.53)	27.3 (1.40)	33.9 (3.83)	27.4 (4.71)
Smoking				
Never	85,608 (59.7%)	101,285 (54.0%)	54,809 (51.5%)	241,702 (55.3%)
Previous	41,891 (29.2%)	67,116 (35.8%)	41,239 (38.8%)	150,246 (34.4%)
Current	15,961 (11.1%)	19,162 (10.2%)	10,322 (9.7%)	45,445 (10.4%)
Alcohol intake				
Daily or almost daily	32,389 (22.6%)	40,452 (21.6%)	16,463 (15.5%)	89,304 (20.4%)
3–4 times a week	35,702 (24.9%)	46,235 (24.7%)	20,550 (19.3%)	10,2487 (23.4%)
Once or twice a week	36,313 (25.3%)	49,273 (26.3%)	28,077 (26.4%)	113,663 (26.0%)
1–3 times a month	14,853 (10.4%)	19,717 (10.5%)	14,346 (13.5%)	48,916 (11.2%)
Special occasions only	14,027 (9.8%)	18,826 (10.0%)	16,405 (15.4%)	49,258 (11.3%)
Never	10,176 (7.1%)	13,060 (7.0%)	10,529 (9.9%)	33,765 (7.7%)
Fruit and vegetable intake (portion/day), mean (SD)	2.01 (0.825)	1.95 (0.827)	1.94 (0.832)	1.97 (0.828)
Red meat (portion/week), mean (SD)	1.93 (1.38)	2.14 (1.42)	2.28 (1.53)	2.11 (1.44)
Processed meat (portion/week), mean (SD)	1.69 (1.08)	1.92 (1.04)	2.03 (1.04)	1.87 (1.06)
Oily fish (portion/week), mean (SD)	1.65 (0.919)	1.65 (0.921)	1.59 (0.946)	1.64 (0.927)
Sedentary time (hours/day), mean (SD)	4.48 (2.03)	5.12 (2.22)	5.64 (2.51)	5.03 (2.28)
Physical activity (hours/day), mean (SD)	1.62 (1.44)	1.76 (1.58)	2.22 (2.00)	1.83 (1.67)
Diabetes at baseline	2398 (1.7%)	7325 (3.9%)	11,485 (10.8%)	21,208 (4.8%)
Hypertension at baseline	20,636 (14.4%)	48,570 (25.9%)	44,758 (42.1%)	11,3964 (26.1%)

Data are presented as numbers (percentages) unless stated otherwise. Participants classified as underweight (BMI < 18.5 kg/m^2^ were excluded from the analyses (*n* = 2629)

*SD* standard deviation, *BMI* body mass index

**Fig. 1:**
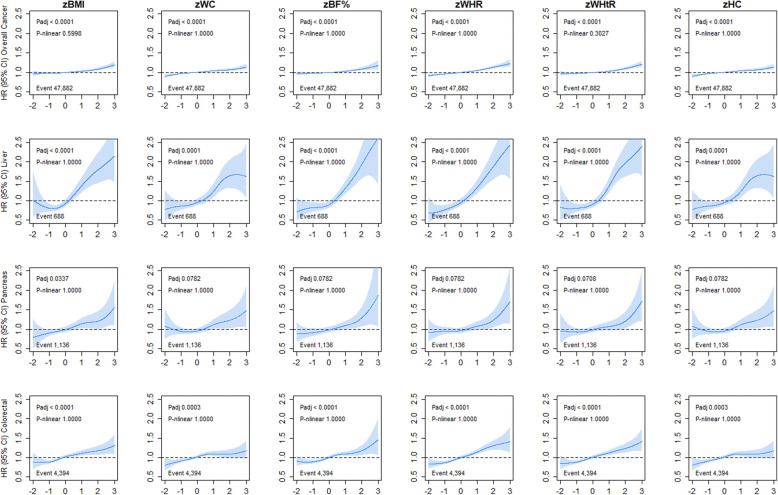
Association of adiposity markers with overall, liver, pancreatic, and colorectal cancer incidence. Penalised splines were used to present the association between adiposity markers and cancer outcomes. The adiposity markers were sex-standardised to 1-SD increment. Analyses were adjusted for age, sex and ethnicity, education, deprivation, smoking, dietary intake (alcohol, fruits and vegetables, red and processed meat, and oily fish), physical activity and sedentary behaviour. BMI, body mass index; BF%, body fat percentage; WHR, waist-hip ratio; WHtR, waist-height ratio; HC, hip circumference; HR, hazard ratio. Shaded areas represent 95% confidence intervals. All *P* values were corrected for multiple testing by using the Holm’s method. Participants classified as underweight (BMI < 18.5 kg/m^2^ were excluded from the analyses (*n* = 2629)

Figure 1 shows the association of six adiposity markers with overall, liver, and colorectal cancer incidence. Although there was no evidence against linear associations with these cancer sites for all adiposity markers, the magnitude of association was higher for liver cancer incidence (HR ranging from 1.19 to 1.33 per 1-SD higher adiposity) compared with colorectal cancer (HR ranging from 1.07 to 1.13 per 1-SD higher adiposity), as shown in Additional file 1: Table S1. Similar results were found for overall, liver, pancreatic, and colorectal cancer mortality as shown in Additional file 1: Table S2. However, the association for WC and HC with colorectal cancer mortality was not significant (Additional file 1: Figure S2). Although a similar shape of association was observed for risk of pancreatic cancer incidence across all adiposity markers, only BMI was significantly associated with a higher risk after adjusting for multiple testing (Fig. 1). Similar results were observed for mortality from pancreatic cancer (Additional file 1: Figure S2). When the analyses were performed by segments of the digestive tract, distal, proximal, and colon cancer incidence were linearly associated with a higher risk across all adiposity markers (Additional file 1: Figure S3), but these associations were not observed for mortality (Additional file 1: Table S1 and Figure S4).

**Fig. 2:**
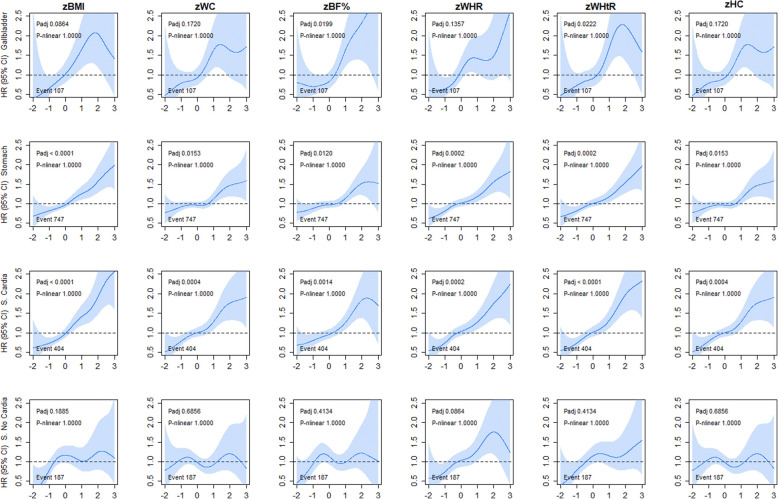
Association of adiposity markers with gallbladder and stomach cancer incidence. Penalised splines were used to present the association between adiposity markers and cancer outcomes. The adiposity markers were sex-standardised to 1-SD increment. Analyses were adjusted for age, sex and ethnicity, education, deprivation, smoking, dietary intake (alcohol, fruits and vegetables, red and processed meat, and oily fish), physical activity and sedentary behaviour. BMI, body mass index; BF%, body fat percentage; WHR, waist-hip ratio; WHtR, waist-height ratio; HC, hip circumference; HR, hazard ratio. Shaded areas represent 95% confidence intervals. All *P* values were corrected for multiple testing by using the Holm’s method. Participants classified as underweight (BMI < 18.5 kg/m^2^ were excluded from the analyses (n = 2629)

The association of adiposity markers with gallbladder and stomach (cardia and non-cardia) cancer incidence is shown in Fig. 2. There was no evidence of non-linear associations for gallbladder cancer across all six adiposity markers (HR varied from 1.28 to 1.50 per 1-SD higher adiposity). For stomach cancer incidence, a linear association was observed across all adiposity markers (HR ranged from 1.14 to 1.24 per 1-SD higher adiposity). However, when the analyses were stratified by stomach cardia and non-cardia, only stomach cardia was linearly associated with all adiposity markers (HR varied from 1.25 to 1.35 per 1-SD higher adiposity) (Additional file 1: Table S1). Similar patterns of associations were observed for mortality from gallbladder, stomach, and stomach cardia cancer (Additional file 1: Figure S5).

**Fig. 3:**
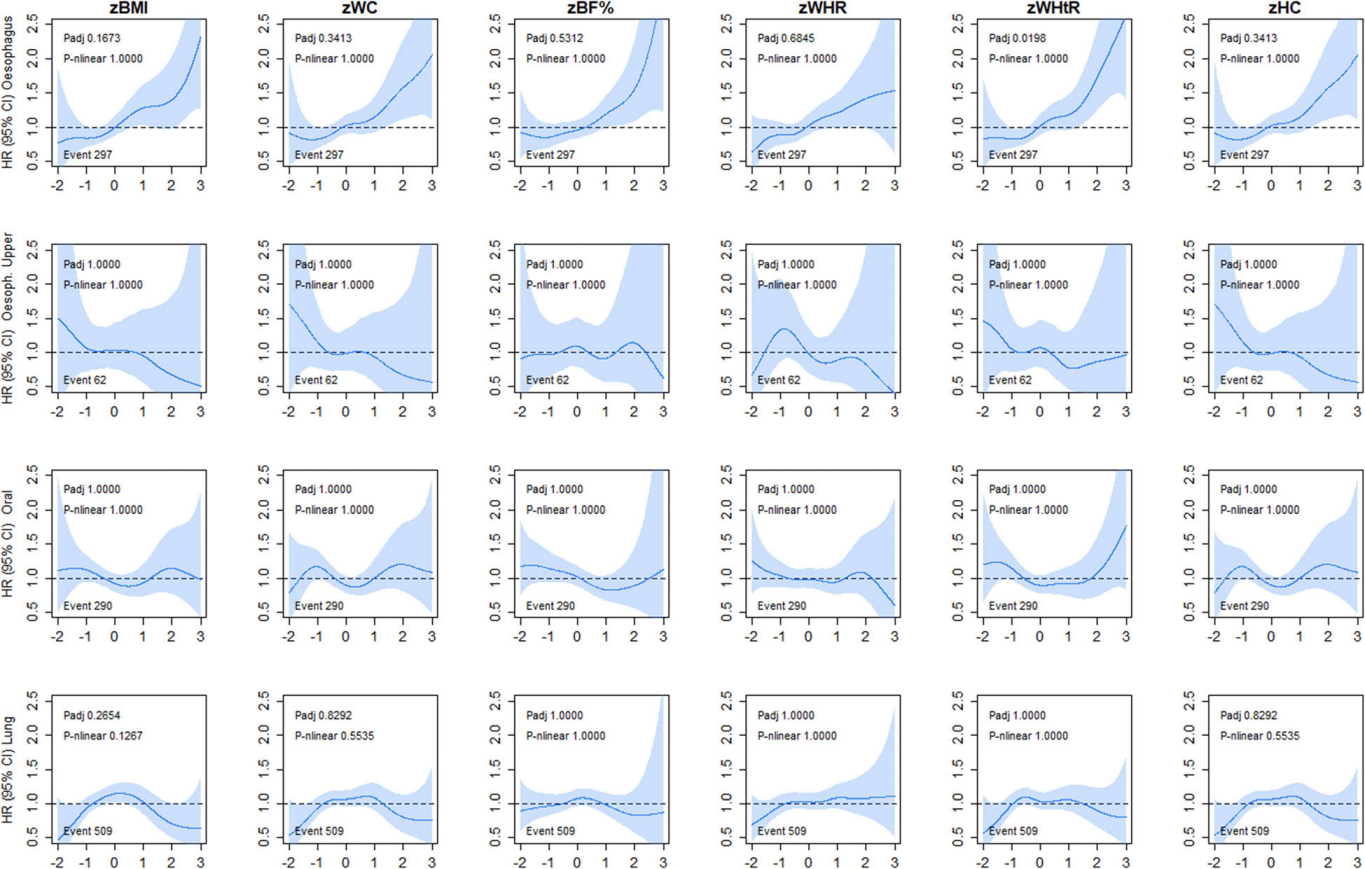
Association of adiposity markers with oesophageal, oral, and lung cancer incidence in never smoker. Penalised splines were used to present the association between adiposity markers and cancer outcomes. The adiposity markers were sex-standardised to 1-SD increment. Analyses were adjusted for age, sex and ethnicity, education, deprivation, dietary intake (alcohol, fruits and vegetables, red and processed meat, and oily fish), physical activity and sedentary behaviour. BMI, body mass index; BF%, body fat percentage; WHR, waist-hip ratio; WHtR, waist-height ratio; HC, hip circumference; HR, hazard ratio. Shaded areas represent 95% confidence intervals. All *P* values were corrected for multiple testing by using the Holm’s method. Participants classified as underweight (BMI < 18.5 kg/m^2^ were excluded from the analyses (n = 2629)

The associations between adiposity and respiratory-related cancers in never smokers are shown in Fig. 3. Although similar shaped associations were observed for oesophageal cancer incidence across all adiposity markers, only WHtR was significant (HR ranged from 1.19 to 1.26 per 1-SD higher adiposity) (Additional file 1: Table S1). Similar associations were observed for oesophageal cancer mortality (Additional file 1: Figure S6). No associations were observed for upper oesophageal, oral, and lung cancer incidence and mortality across any of the adiposity markers.

**Fig. 4:**
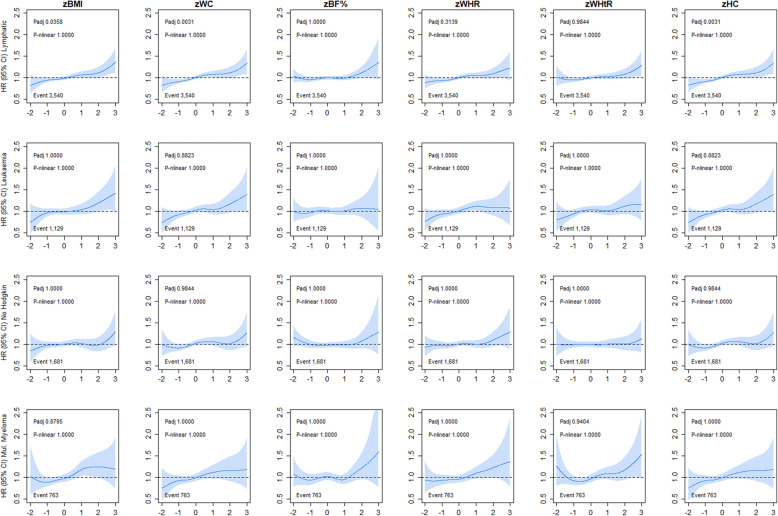
Association of adiposity markers with lymphatic cancer incidence. Penalised splines were used to present the association between adiposity markers and cancer outcomes. The adiposity markers were sex-standardised to 1-SD increment. Analyses were adjusted for age, sex and ethnicity, education, deprivation, smoking, dietary intake (alcohol, fruits and vegetables, red and processed meat, and oily fish), physical activity and sedentary behaviour. BMI, body mass index; BF%, body fat percentage; WHR, waist-hip ratio; WHtR, waist-height ratio; HC, hip circumference; HR, hazard ratio. Shaded areas represent 95% confidence intervals. All *P* values were corrected for multiple testing by using the Holm’s method. Participants classified as underweight (BMI < 18.5 kg/m^2^ were excluded from the analyses (n = 2629)

Lymphatic cancer was linearly associated with BMI, WC, and HC, for incidence (HR ranged from 1.06 to 1.08 per 1-SD higher adiposity, Additional file 1: Table S1). However, no association were observed for leukaemia, non-Hodgkin and myeloma cancer incidence and mortality across any of the adiposity markers (Fig. 4 and Additional file 1: Figure S7).

**Fig. 5:**
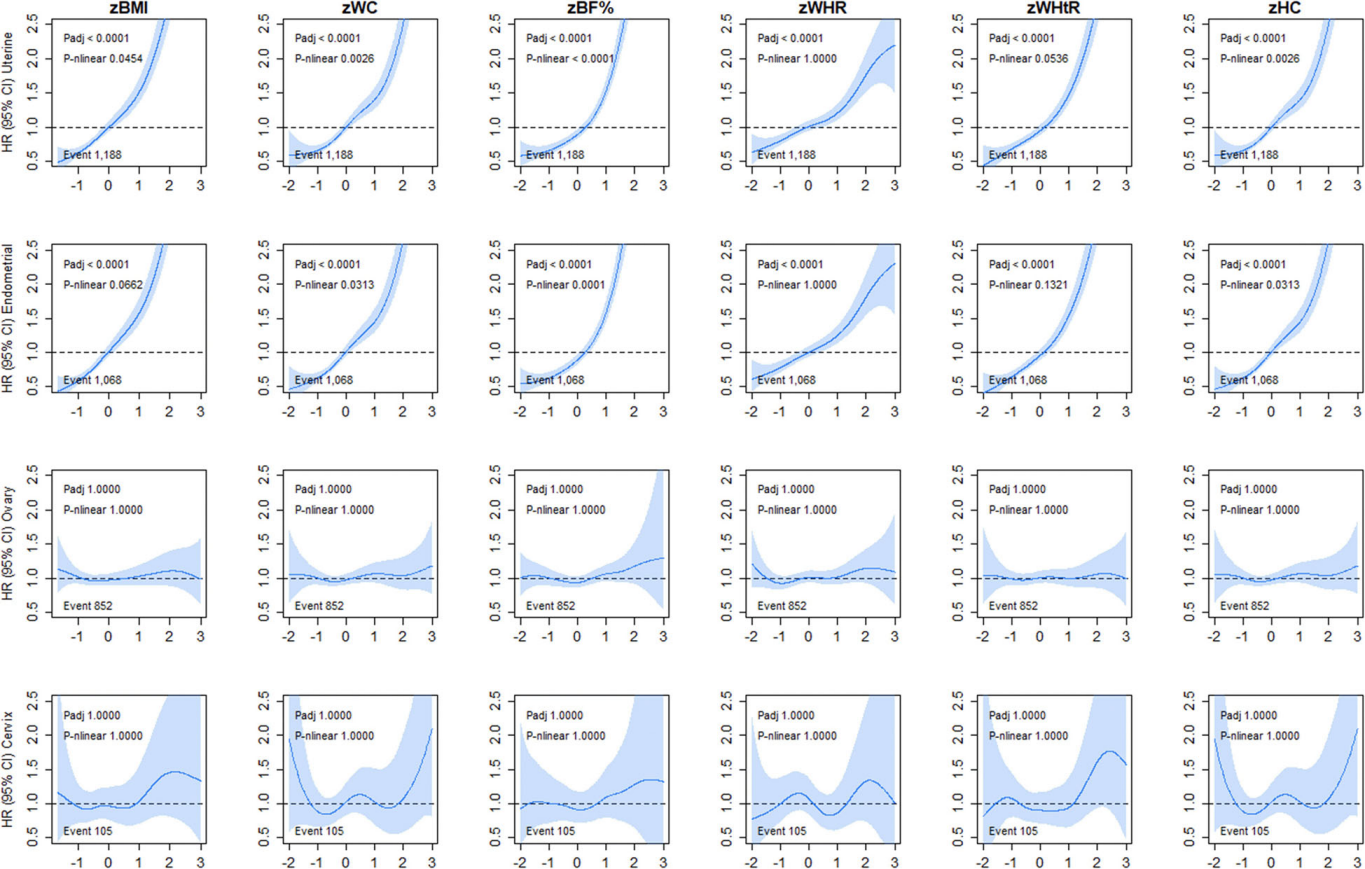
Association of adiposity markers with women-specific cancer incidence. Penalised splines were used to present the association between adiposity markers and cancer outcomes. The adiposity markers were sex-standardised to 1-SD increment. Analyses were adjusted for age, sex and ethnicity, education, deprivation, smoking, dietary intake (alcohol, fruits and vegetables, red and processed meat, and oily fish), physical activity and sedentary behaviour. BMI, body mass index; BF%, body fat percentage; WHR, waist ratio; WHtR, waist-height ratio; HC, hip circumference; HR, hazard ratio. Shaded areas represent 95% confidence intervals. All *P* values were corrected for multiple testing by using the Holm’s method. Participants classified as underweight (BMI < 18.5 kg/m^2^ were excluded from the analyses (n = 2629)

**Fig. 6:**
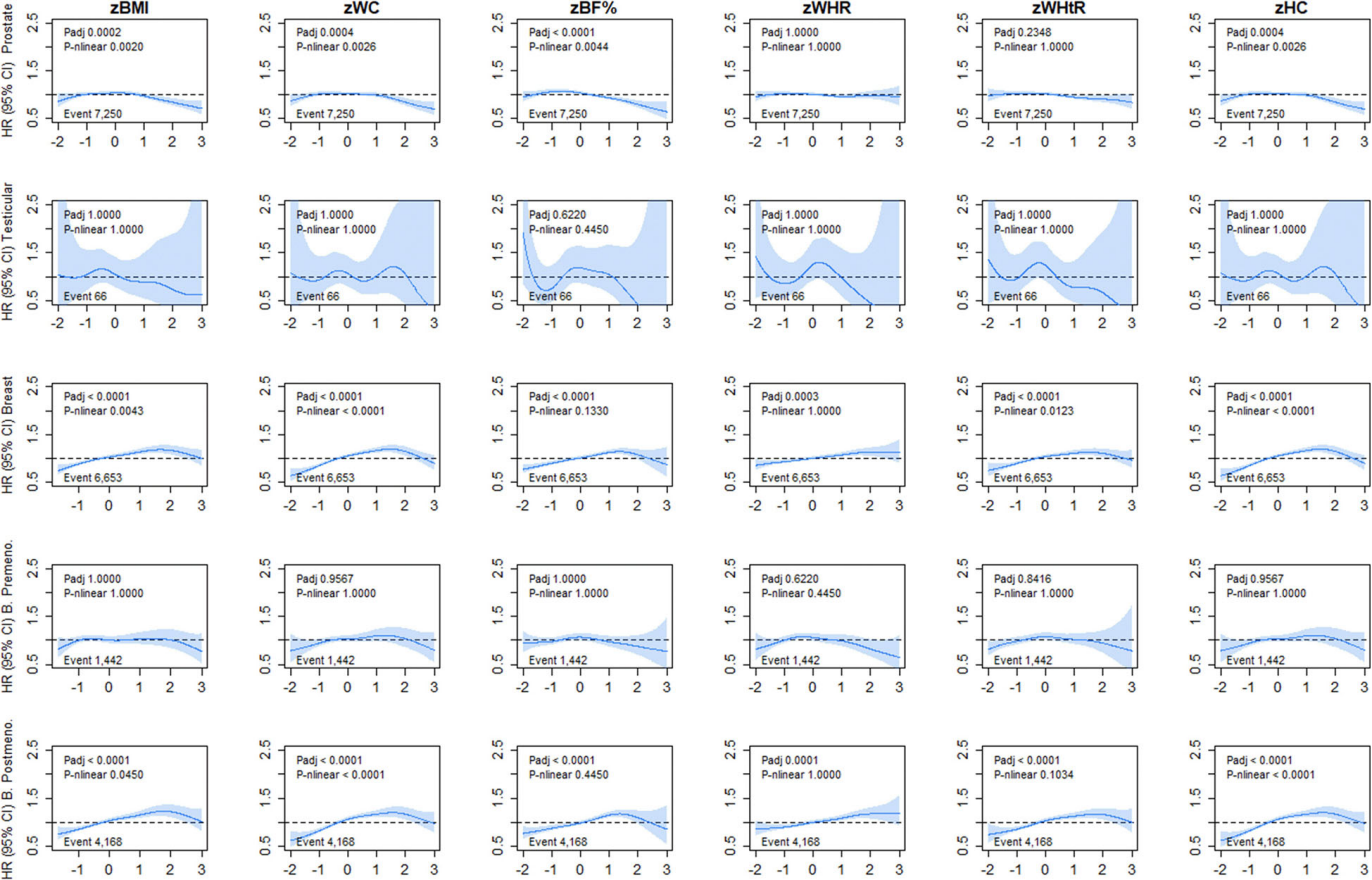
Association of adiposity markers with prostate, testicular cancer in men and breast cancer (overall, pre and post menopausal) incidence. Penalised splines were used to present the association between adiposity markers and cancer outcomes. The adiposity markers were sex-standardised to 1-SD increment. Analyses were adjusted for age, sex and ethnicity, education, deprivation, smoking, dietary intake (alcohol, fruits and vegetables, red and processed meat, and oily fish), physical activity and sedentary behaviour. BMI, body mass index; BF%, body fat percentage; WHR, waist-hip ratio; WHtR, waist-height ratio; HC, hip circumference; HR, hazard ratio. Shaded areas represent 95% confidence intervals. All *P* values were corrected for multiple testing by using the Holm’s method. Participants classified as underweight (BMI < 18.5 kg/m^2^ were excluded from the analyses (n = 2629)

For sex-specific cancers, we observed a steeper linear association for uterine (HR ranged from 1.26 to 1.70) and endometrial (HR ranged from 1.29 to 1.78) cancer incidence (Fig. 5 and Additional file 1: Table S1). The strongest magnitude of association for both uterine and endometrial cancer incidence was observed for BF% whereas WHR shows the smallest magnitude of association of any adiposity marker (Additional file 1: Table S1). Although similar associations were observed for uterine and endometrial cancer mortality across all adiposity markers, mortality from cervical cancer showed a borderline U-shaped association with BMI, WC, BF%, WHtR, and HC (Additional file 1: Figure S9 and Table S2). No association was found between adiposity and ovarian cancer incidence and mortality. For breast cancer incidence, a linear association was observed for BMI, BF%, WHtR, and WHR; however, a slight departure from linearity was observed for WC and HC (Fig. 6). When the analyses were stratified into pre and post menopause, the adiposity markers were associated with breast cancer incidence in postmenopausal women only (Fig. 6). No associations were observed for breast cancer mortality (Additional file 1: Figure S10). The associations for women-related cancers remained largely unchanged when the analyses were further adjusted for use of hormonal replacement therapy, age at menarche, and age at first and date of last live birth (Additional file 1: Figure S8, S9 and S11). For men, only prostate cancer incidence, but not mortality, was inversely associated with WC and HC (Fig. 6 and Additional file 1: Figure S10).

**Fig. 7:**
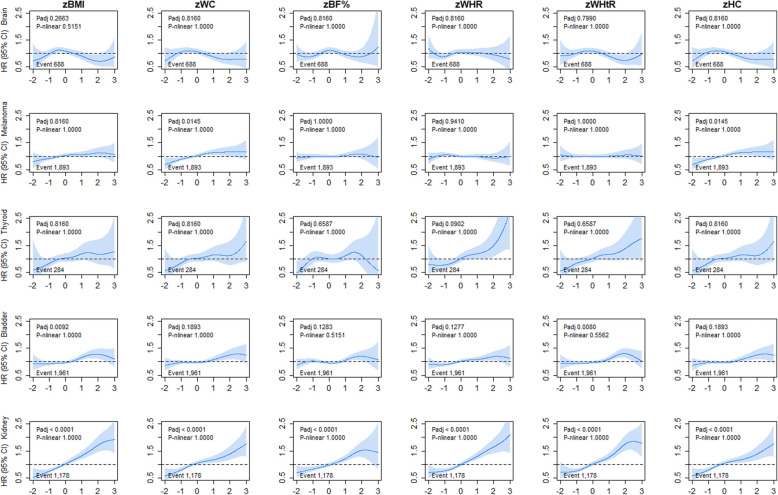
Association of adiposity markers with brain, melanoma, thyroid, bladder, and kidney cancer incidence. Penalised splines were used to present the association between adiposity markers and cancer outcomes. The adiposity markers were sex-standardised to 1-SD increment. Analyses were adjusted for age, sex and ethnicity, education, deprivation, smoking, dietary intake (alcohol, fruits and vegetables, red and processed meat, and oily fish), physical activity and sedentary behaviour. BMI, body mass index; BF%, body fat percentage; WHR, waist-hip ratio; WHtR, waist-height ratio; HC, hip circumference; HR, hazard ratio. Shaded areas represent 95% confidence intervals. All *P* values were corrected for multiple testing by using the Holm’s method. Participants classified as underweight (BMI < 18.5 kg/m^2^ were excluded from the analyses (n = 2629)

Kidney cancer incidence and mortality were linearly associated with all adiposity markers, with HR ranging from 1.18 to 1.27 per 1-SD higher adiposity (Fig. 7 and Additional file 1: Figure S12 and Table S1). For bladder cancer, we observed a higher risk of cancer incidence only at the higher end of the BMI and WHtR ranges (Fig. 7). However, these associations were not observed for bladder cancer mortality (Additional file 1: Figure S12). For melanoma cancer incidence, only WC and HC were linearly associated with a higher risk (Fig. 7).

**Fig. 8:**
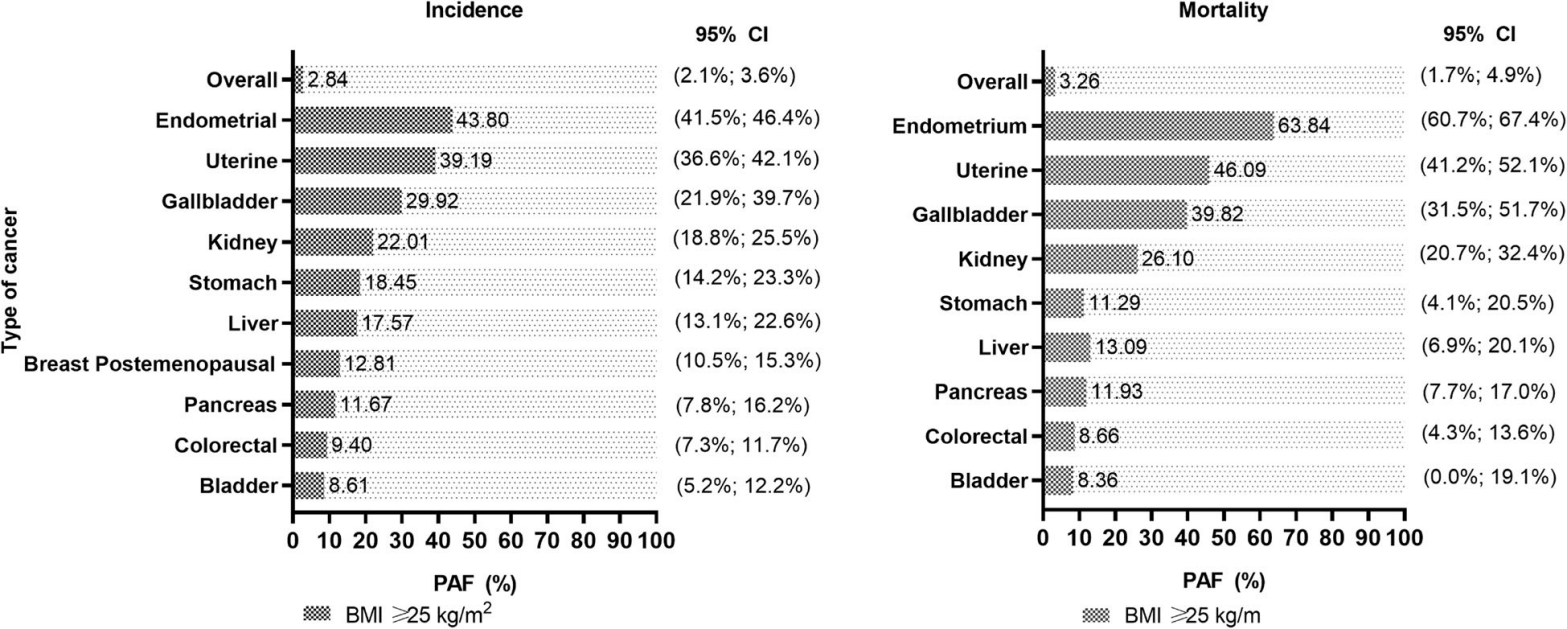
Population attributable fraction (PAF) for cancer incidence and mortality attributable to have a BMI ≥ 25 kg/m^2^. Data are presented in percentages. Analyses were adjusted for age, sex and ethnicity, education, deprivation, smoking, dietary intake (alcohol, fruits and vegetables, red and processed meat, and oily fish), physical activity and sedentary behaviour. Breast cancer was additionally adjusted for age at menarche, hormonal replacement, and age at first and last live birth. Normal BMI (18.5 to 24.9 kg/m^2^) was used as the reference group and compared with individuals with BMI ≥ 25 kg/m^2^)

Our PAF analyses show that the proportions of cancer attributable to BMI vary considerably by cancer site. Endometrial, uterine, and gallbladder were the top three cancers for which obesity accounted for 43.8%, 39.2%, and 29.9% incident cases and 63.8%, 46.1%, and 39.8% of deaths, respectively (Fig. 8). When the predictive ability of BMI was compared with the other adiposity markers using C-index, there were no evidence of a significant improvement in C-indices from models using WC, BF%, WHR, WHtR, and HC over the model with BMI (Additional file 1: Table S3). The associations for overall, liver, kidney, stomach, pancreatic, bladder, gallbladder, colorectal cancer, endometrium, uterine, and breast (postmenopausal in women) cancer remained significant and largely unchanged when the analyses were adjusted for competing events (Additional file 1: Table S4).

When we conducted the analyses including underweight people, the association between adiposity and cancer remained linear (Additional file 1: Figure S13 and Figure S14). Similar results were found for cancer incidence and mortality when we added height as a covariate; some associations were slightly stronger as was the association between BMI and overall cancer incidence and mortality; (Additional file 1: Table S5 and Table S6).

## Discussion

This study provides important evidence regarding the risk of 24 cancer sites associated with multiple adiposity markers. Higher levels of adiposity, regardless of the adiposity marker used, were associated in a linear manner with a higher incidence of liver, kidney, stomach, pancreatic, bladder, gallbladder, colorectal cancer, endometrial, uterine, and breast (in postmenopausal women) cancer. If the associations observed were causal, reducing the BMI of obese individuals to the normal range could prevent 43.8%, 39.2%, and 29.9% of incidence and 63.8%, 46.1%, and 39.8% deaths from endometrial, uterine, and gallbladder cancers, respectively.

Our findings corroborate previous evidence, including the WCRF obesity and cancer 2018 report and meta-analyses from protective cohort studies [31–34], that adult adiposity (assessed using BMI) is associated with higher risk of oesophageal, pancreatic, liver, colorectal, postmenopausal breast, and endometrial cancers. Furthermore, our findings add strength to previously weak evidence of links between BMI and stomach cancer risk [35]. On the other hand, our findings did not find evidence for an association between BMI (and any other markers of adiposity) and ovarian cancer as reported by others [36], which could be attributed to our comprehensive confounder adjustments. We also found inverse associations between five adiposity markers and risk of prostate cancer. Although excess adiposity has been associated with multiple cancers, evidence of its association with prostate cancer has been restricted to advanced prostate cancer only [37, 38]. However, a recent systematic review of data from 78 studies, including a meta-analysis of 67 studies, reported no association between BMI and prostate cancer [37, 38]. These authors also concluded that previously reported inverse associations between BMI and prostate cancer may be due to incomplete diagnosis (not all men being biopsied). The assumption that men who have not been tested for prostate do not have prostate cancer may lead to bias and inverse associations [37]. BMI and WHtR were positively associated with bladder cancer, in concordance with the meta-analysis of 15 cohort studies, published by Sun et al., which showed a linear association between adiposity and bladder cancer [39].

We did not find a significant association between adiposity and lung cancer in never smokers. These disagree with a recent meta-analysis with considerable statistical power, which pooled data from 29 observational studies, including 15 million never smokers, where BMI was inversely associated with lung cancer [40]. 

There is convincing evidence [41] that greater adiposity is associated with increased risk of colorectal cancer, assessed mainly as BMI in prospective cohort studies [7, 35, 41–44]. Our study corroborates these findings and adds novel evidence that other adiposity markers are also consistently associated with an increased risk of colorectal cancer. We also observed that all adiposity markers were positively associated with higher liver cancer risk with broadly consistent effect sizes. Furthermore, we found that all adiposity markers were associated with an increased risk of breast cancer. But the association appeared to occur in postmenopausal women only. These findings confirm previous evidence from prospective cohort studies [33, 45, 46].

### Implications of findings

The findings of this study have important clinical implications. First, it provides evidence that central (waist and hip circumference) and overall adiposity (BMI and BF%) markers produced similar relative risk estimates. Therefore, the use of BMI, a simple and low-cost measurement, is adequate for clinical screening in terms of cancer risk, and there is no advantage in using more complicated or more expensive measures such as WC or BF%. We also found that a significant proportion of cancers could be prevented by reducing obesity, especially liver and kidney cancer in men and endometrial and uterine cancer in women.

### Strengths and Limitations

UK Biobank is not a representative sample of the UK older adult population, so we should be cautious in generalising summary statistics to the general population. However, relative risks derived from UK Biobank are consistent with more representative population cohorts [47]. The adiposity exposures used in the study were measured by trained staff using standardised protocols; therefore, this minimises the chance of measurement error and misclassification. However, there are several limitations that should be taken into account. Reverse causation is a concern in prospective cohort studies investigating the association between adiposity and cancer. However, to minimise the effect of reverse causation in our study, we excluded all participants with cancers diagnosed within the first 2 years of follow-up. Residual confounding is also possible even though we have adopted a comprehensive adjustment scheme. In addition, although we used data from hospital admission and deaths registers, available in the UK, we cannot exclude misclassification for cancer-specific sites or uncommon cancers. Although UK Biobank is a large observational study, some cancers had limited numbers of events, which limited our power to identify some associations with adiposity markers.

## Conclusion

Adiposity, regardless of the marker used, was associated with an increased risk of 10 cancer sites. Furthermore, the associations were mostly linear among all adiposity markers. We found no evidence that the use of other adiposity markers, such as central adiposity or body fat, improves the prediction ability for cancer risk beyond the risk attributable to BMI.

## Supplementary information

**Additional file 1:** **Figure S1.** Formula for PAFs. **Figure S2.** Association of Adiposity markers with overall, liver, pancreas and colorectal cancer mortality. **Figure S3.** Association of Adiposity markers with colorectal cancer incidence. **Figure S4.** Association of Adiposity markers with colorectal cancer mortality. **Figure S5.** Association of Adiposity markers with gallbladder and stomach cancer mortality. **Figure S6.** Association of Adiposity markers oesophagus, oral and lung cancer mortality in no smokers. **Figure S7.** Association of Adiposity markers with lymphatic cancer mortality. **Figure S8.** Association of Adiposity markers with uterine, endometrial, ovary and cervical cancer incidence adjusted. **Figure S9.** Association of Adiposity markers with uterine, endometrial, ovary and cervical cancer mortality. **Figure S10.** Association of Adiposity markers with prostate, testicular cancer in men and breast cancer in postmenopausal women mortality. **Figure S11.** Association of adiposity markers with prostate, testicular, and breast cancer incidence additionally adjusted for sex-related covariates. **Figure S12.** Association of adiposity markers with brain, melanoma, thyroid, bladder and kidney cancer mortality. **Figure S13.** Association of Adiposity markers with overall, liver, pancreas, colorectal cancer and stomach cardia incidence with underweight people. **Figure S14.** Association of Adiposity markers with gallbladder, bladder, kidney, breast and endometrium cancer incidence with underweight people. **Table S1.** Association of adiposity markers with incidence from 24 cancer sites per 1 SD increase in adiposity markers. **Table S2.** Association of adiposity markers with mortality from 24 cancer sites per 1 SD increase in adiposity markers. **Table S3.** C-Index for the predictive ability of BMI versus other adiposity markers. **Table S4.** Association of adiposity markers with incidence from 24 cancer sites after accounting for competing risk. **Table S5.** Association of adiposity markers with incidence from 24 cancer sites per 1 SD increase in adiposity markers with height as covariate. **Table S6.** Association of adiposity markers with mortality from 24 cancer sites per 1 SD increase in adiposity markers with height as covariate.

## Data Availability

The data that support the findings of this study are available from UK Biobank but restrictions apply to their availability. These data were used under licence for the current study and so are not publicly available. The data are, however, available from the authors upon reasonable request and with permission of UK Biobank.

## Abbreviations

95% CI: 95% confidence intervals
BF%: Body fat percentage
BFI: Body fat mass index
BMI: Body mass index
HC: Hip circumference
HR: Hazard ratio
Padj: *P* value for linear association adjusted for multiple testing
P-nlinear: *P* value for non-linear association adjusted for multiple testing
SD: Standard deviation
WC: Waist circumference
WHR: Waist-hip ratio
WHtR: Waist-height ratio
