# Supplementary material for: Associations of six adiposity-related markers with incidence and mortality from 24 cancers—findings from the UK Biobank prospective cohort study
Source: BMC Med. 2021 Jan 11;19:7. doi: 10.1186/s12916-020-01848-8 (PMC7798245; doi:10.1186/s12916-020-01848-8)
Supplement: Supplementary file 1 — Additional file 1: Figure S1. Formula for PAFs. Figure S2. Association of Adiposity markers with overall, liver, pancreas and colorectal cancer mortality. Figure S3. Association of Adiposity markers with colorectal cancer incidence. Figure S4. Association of Adiposity markers with colorectal cancer mortality. Figure S5. Association of Adiposity markers with gallbladder and stomach cancer mortality. Figure S6. Association of Adiposity markers oesophagus, oral and lung cancer mortality in no smokers. Figure S7. Association of Adiposity markers with lymphatic cancer mortality. Figure S8. Association of Adiposity markers with uterine, endometrial, ovary and cervical cancer incidence adjusted. Figure S9. Association of Adiposity markers with uterine, endometrial, ovary and cervical cancer mortality. Figure S10. Association of Adiposity markers with prostate, testicular cancer in men and breast cancer in postmenopausal women mortality. Figure S11. Association of adiposity markers with prostate, testicular, and breast cancer incidence additionally adjusted for sex-related covariates. Figure S12. Association of adiposity markers with brain, melanoma, thyroid, bladder and kidney cancer mortality. Figure S13. Association of Adiposity markers with overall, liver, pancreas, colorectal cancer and stomach cardia incidence with underweight people. Figure S14. Association of Adiposity markers with gallbladder, bladder, kidney, breast and endometrium cancer incidence with underweight people. Table S1. Association of adiposity markers with incidence from 24 cancer sites per 1 SD increase in adiposity markers. Table S2. Association of adiposity markers with mortality from 24 cancer sites per 1 SD increase in adiposity markers. Table S3. C-Index for the predictive ability of BMI versus other adiposity markers. Table S4. Association of adiposity markers with incidence from 24 cancer sites after accounting for competing risk. Table S5. Association of adiposity markers with incidence from [file 12916_2020_1848_MOESM1_ESM.docx]

**Additional file 1: Non-linear associations of six adiposity-related markers with incidence and mortality from 24 cancer sites in the UK Biobank prospective cohort study.**

****Figures with red lines are for mortality; those with blue lines for incidence.**

**Figure S1: Formula** Population Attributable Fraction


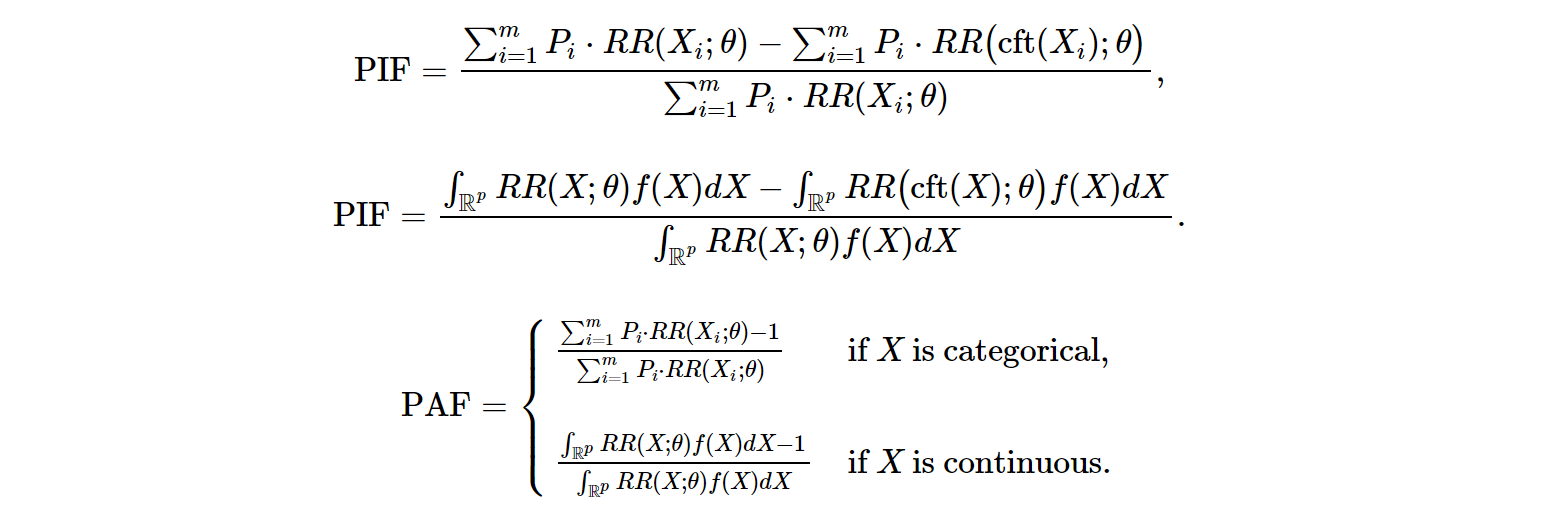


**PAF =**

PAF: Population Attributable Fraction

RR(X_i_;θ): Relative Risk

X_i_: i^th^ category of the exposure variable

**
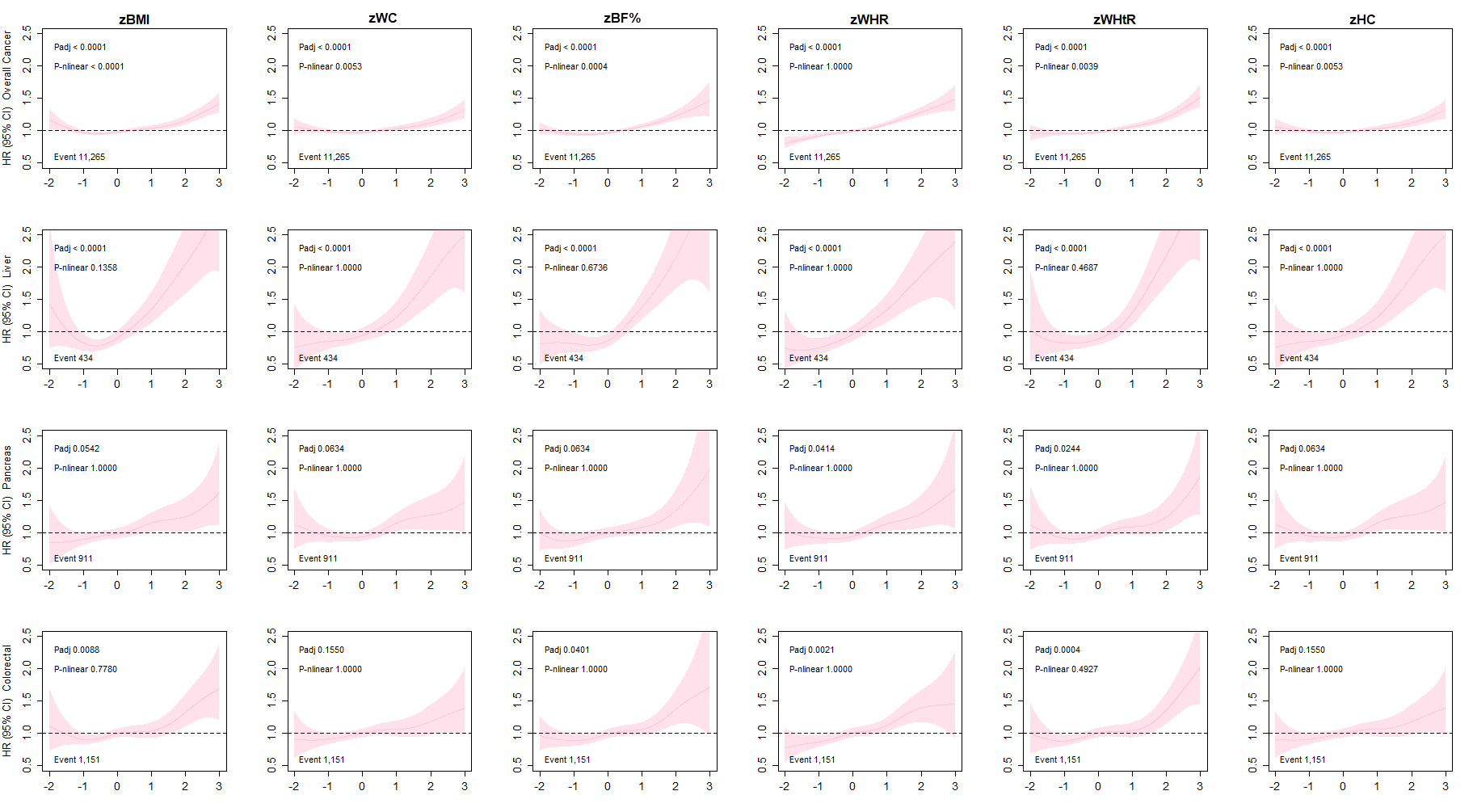
**

**Figure S2.** Association of adiposity markers with overall, liver, pancreas and colorectal cancer mortality.

Penalised splines were used to present the association between adiposity markers and cancer outcomes. The adiposity markers were sex-standardised to 1-SD increment. Analyses were adjusted age, sex, ethnicity, education, deprivation, smoking, dietary intake (alcohol, fruits & vegetables, red & processed meat, and oily fish), discretionary sedentary behaviour and physical activity. BMI: Body Mass Index, BF%: Body Fat Percentage, WHR: waits hip ratio, WHTR: Waist height ratio, HC: hip circumference, HR: Hazard Ratio. Shaded areas represent 95% confidence intervals. P-value for linear association corrected for multiple testing (Padj), p-value for non-linear association corrected for multiple testing (P-nlinear). Participants classified as underweight (BMI < 18.5 kg/m2 were excluded from the analyses (n = 2629).

**
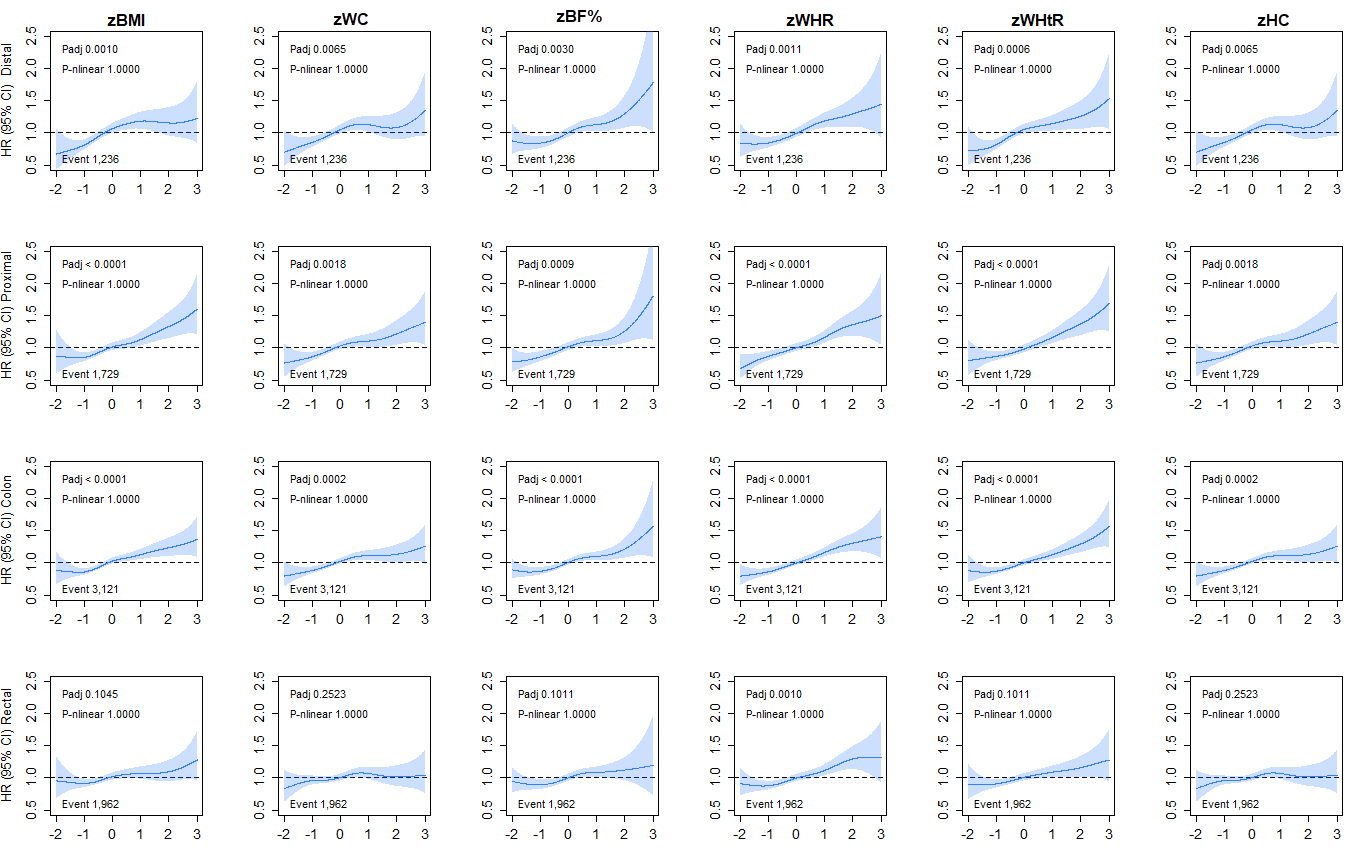
**

**Figure S3.** Association of adiposity markers with distal, proximal, colon and rectal cancer incidence.

Penalised splines were used to present the association between adiposity markers and cancer outcomes. The adiposity markers were sex-standardised to 1-SD increment. Analyses were adjusted age, sex, ethnicity, education, deprivation, smoking, dietary intake (alcohol, fruits & vegetables, red & processed meat, and oily fish), discretionary sedentary behaviour and physical activity. BMI: Body Mass Index, BF%: Body Fat Percentage, WHR: waits hip ratio, WHTR: Waist height ratio, HC: hip circumference, HR: Hazard Ratio. Shaded areas represent 95% confidence intervals. P-value for linear association corrected for multiple testing (Padj), p-value for non-linear association corrected for multiple testing (P-nlinear). Participants classified as underweight (BMI < 18.5 kg/m2 were excluded from the analyses (n = 2629).

**
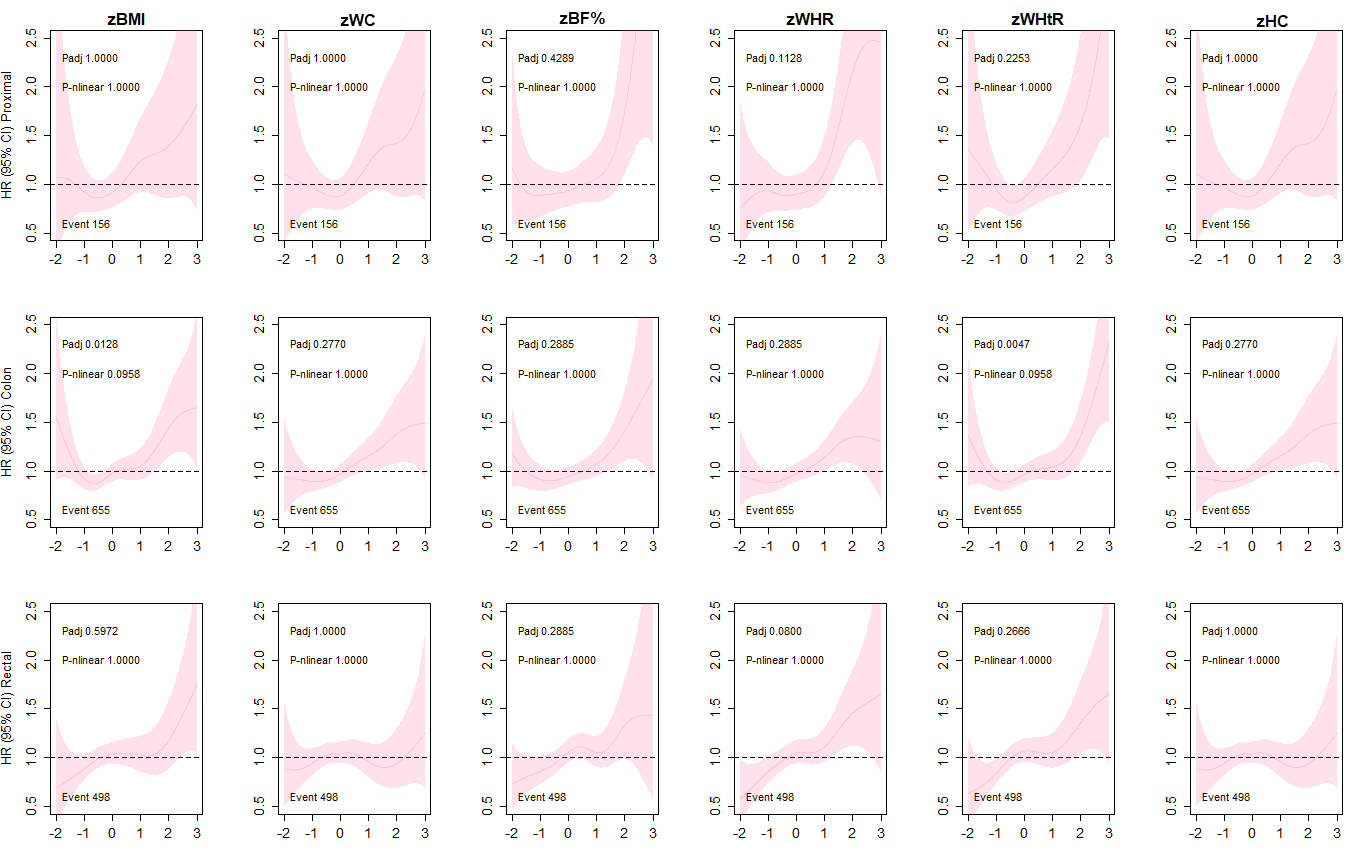
**

**Figure S4.** Association of adiposity markers with proximal, colon and rectal cancer mortality.

Penalised splines were used to present the association between adiposity markers and cancer outcomes. The adiposity markers were sex-standardised to 1-SD increment. Analyses were adjusted age, sex, ethnicity, education, deprivation, smoking, dietary intake (alcohol, fruits & vegetables, red & processed meat, and oily fish), discretionary sedentary behaviour and physical activity. BMI: Body Mass Index, BF%: Body Fat Percentage, WHR: waits hip ratio, WHTR: Waist height ratio, HC: hip circumference, HR: Hazard Ratio. Shaded areas represent 95% confidence intervals. P-value for linear association corrected for multiple testing (Padj), p-value for non-linear association corrected for multiple testing (P-nlinear). Participants classified as underweight (BMI < 18.5 kg/m2 were excluded from the analyses (n = 2629).


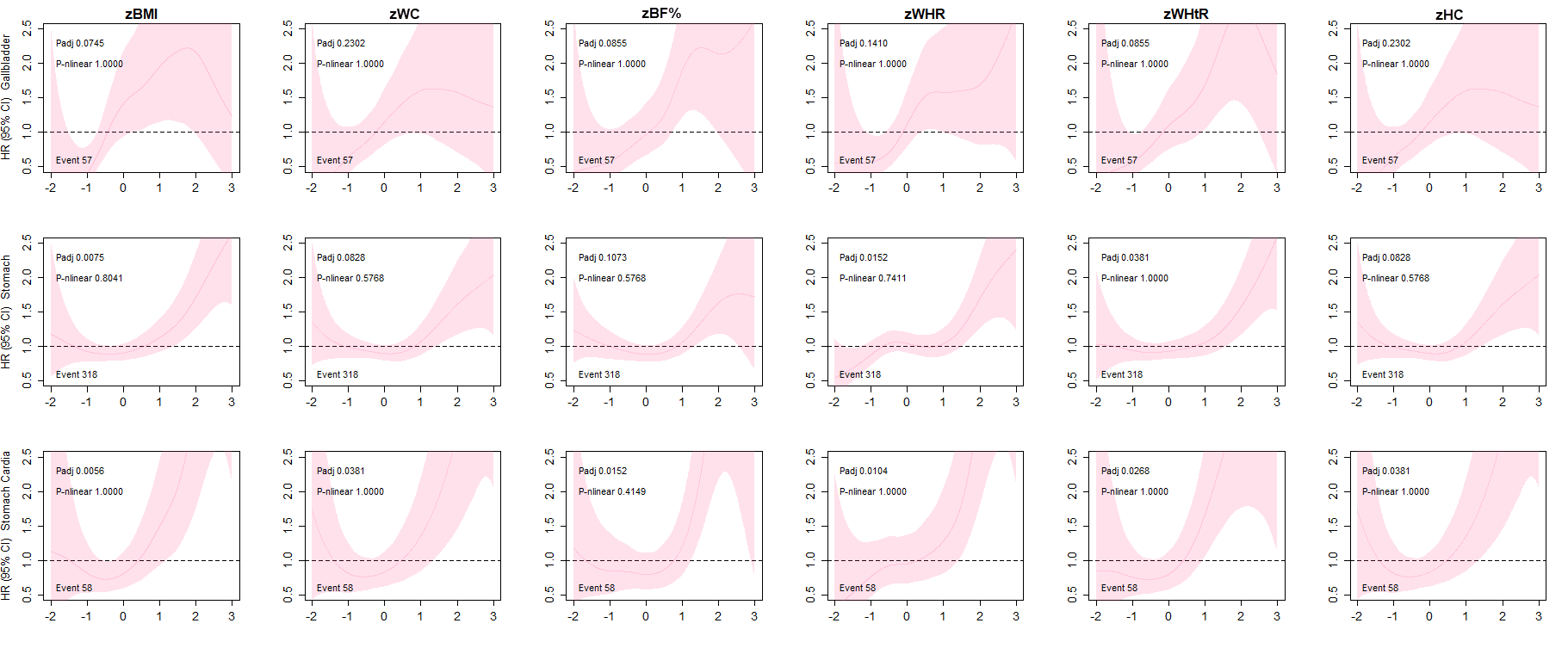


**Figure S5.** Association of adiposity markers with gallbladder and stomach cancer mortality.

Penalised splines were used to present the association between adiposity markers and cancer outcomes. The adiposity markers were sex-standardised to 1-SD increment. Analyses were adjusted age, sex, ethnicity, education, deprivation, smoking, dietary intake (alcohol, fruits & vegetables, red & processed meat, and oily fish), discretionary sedentary behaviour and physical activity. BMI: Body Mass Index, BF%: Body Fat Percentage, WHR: waits hip ratio, WHTR: Waist height ratio, HC: hip circumference, HR: Hazard Ratio. Shaded areas represent 95% confidence intervals. Stomach no cardia did not have enough cases to perform the analyses for mortality (n>5). P-value for linear association corrected for multiple testing (Padj), p-value for non-linear association corrected for multiple testing (P-nlinear). Participants classified as underweight (BMI < 18.5 kg/m2 were excluded from the analyses (n = 2629).


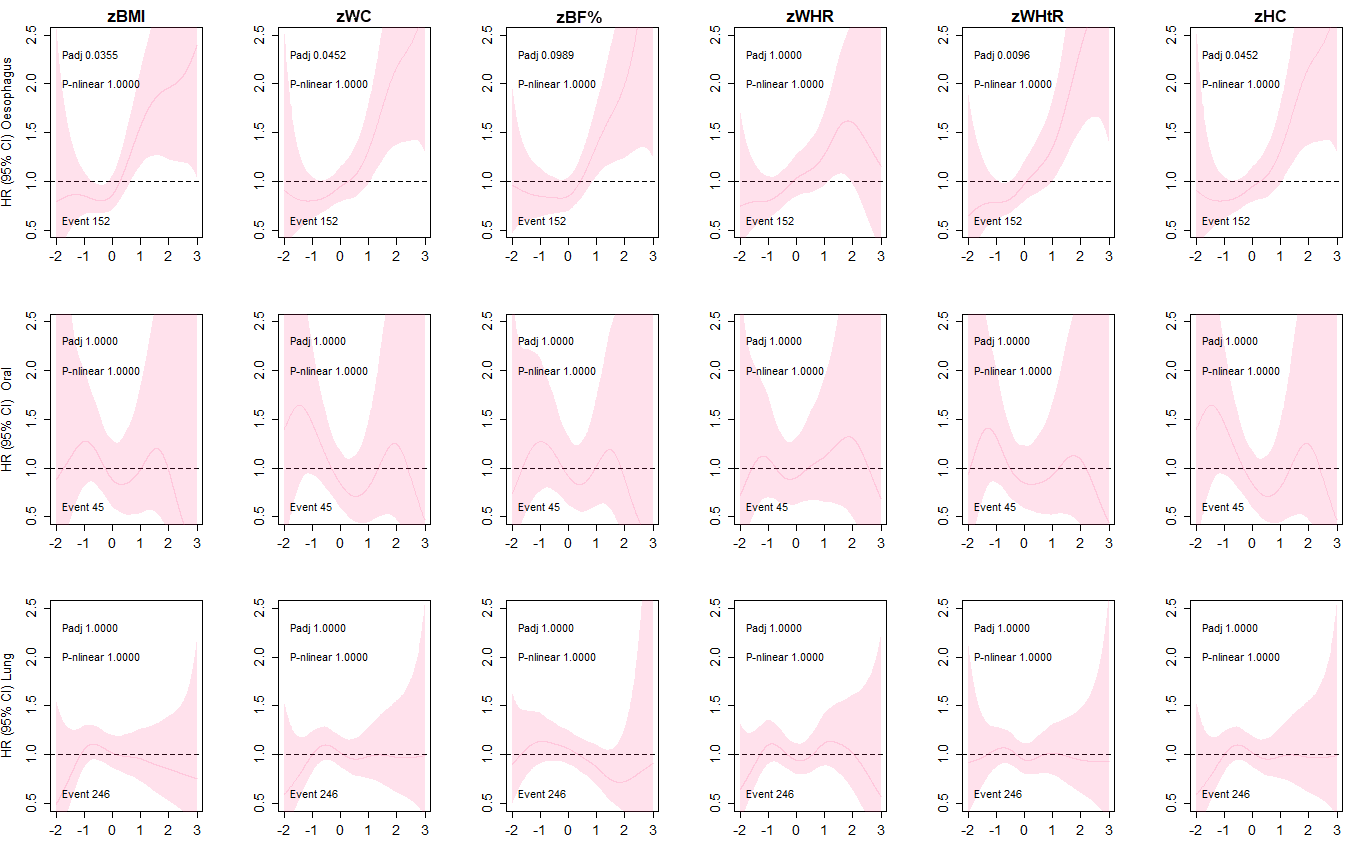


**Figure S6.** Association of adiposity markers oesophagus, oral and lung cancer mortality in never smokers.

Penalised splines were used to present the association between adiposity markers and cancer outcomes. The adiposity markers were sex-standardised to 1-SD increment. Analyses were adjusted age, sex, ethnicity, education, deprivation, dietary intake (alcohol, fruits & vegetables, red & processed meat, and oily fish), discretionary sedentary behaviour and physical activity. BMI: Body Mass Index, BF%: Body Fat Percentage, WHR: waits hip ratio, WHTR: Waist height ratio, HC: hip circumference, HR: Hazard Ratio. Shaded areas represent 95% confidence intervals. Oesophagus upper did not have enough cases to perform the mortality analysis (n>5). P-value for linear association corrected for multiple testing (Padj), p-value for non-linear association corrected for multiple testing (P-nlinear). Participants classified as underweight (BMI < 18.5 kg/m2 were excluded from the analyses (n = 2629).


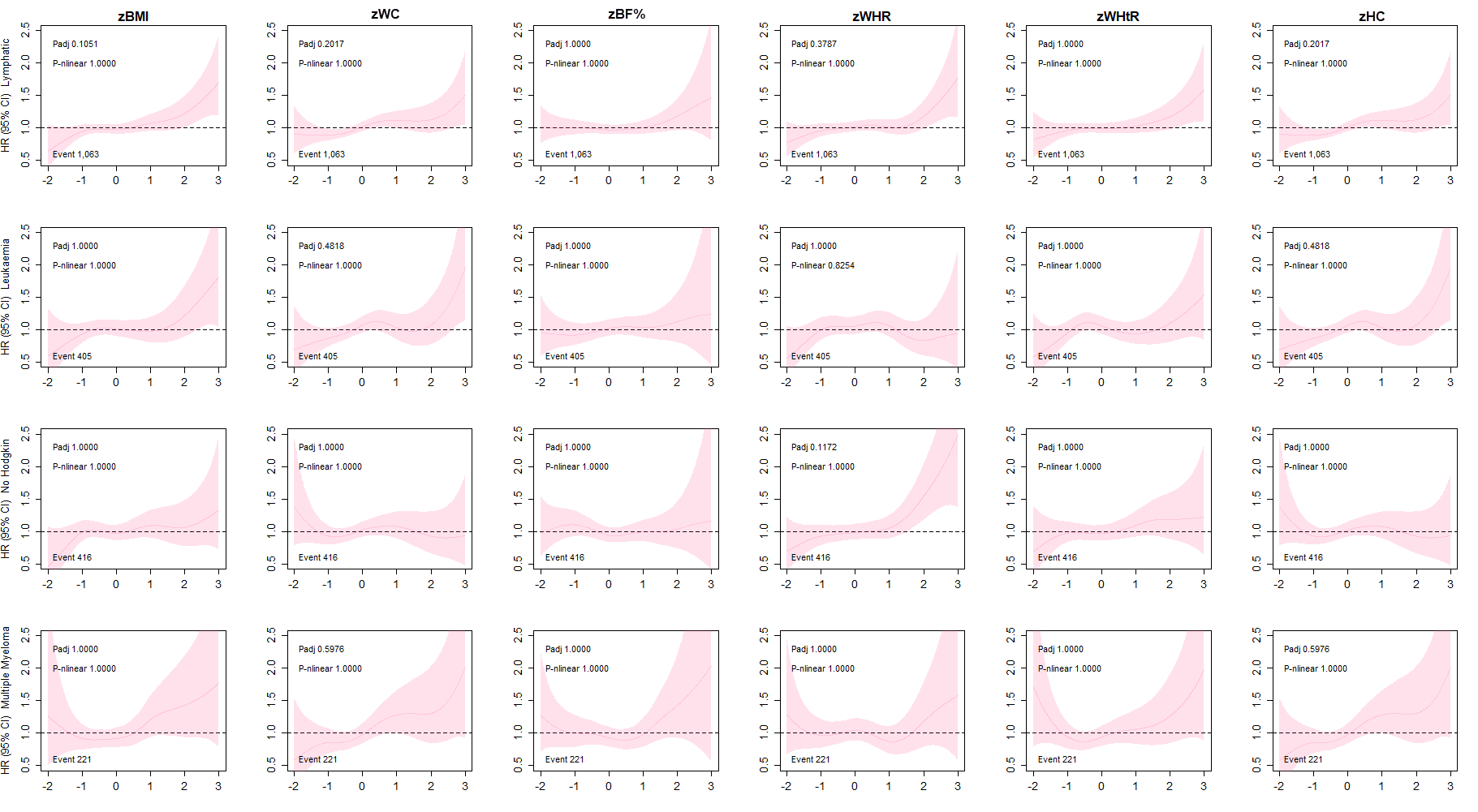


**Figure S7.** Association of adiposity markers with lymphatic cancer mortality.

Penalised splines were used to present the association between adiposity markers and cancer outcomes. The adiposity markers were sex-standardised to 1-SD increment. Analyses were adjusted age, sex, ethnicity, education, deprivation, smoking, dietary intake (alcohol, fruits & vegetables, red & processed meat, and oily fish), discretionary sedentary behaviour and physical activity. BMI: Body Mass Index, BF%: Body Fat Percentage, WHR: waits hip ratio, WHTR: Waist height ratio, HC: hip circumference, HR: Hazard Ratio. Shaded areas represent 95% confidence intervals. P-value for linear association corrected for multiple testing (Padj), p-value for non-linear association corrected for multiple testing (P-nlinear). Participants classified as underweight (BMI < 18.5 kg/m2 were excluded from the analyses (n = 2629).

**
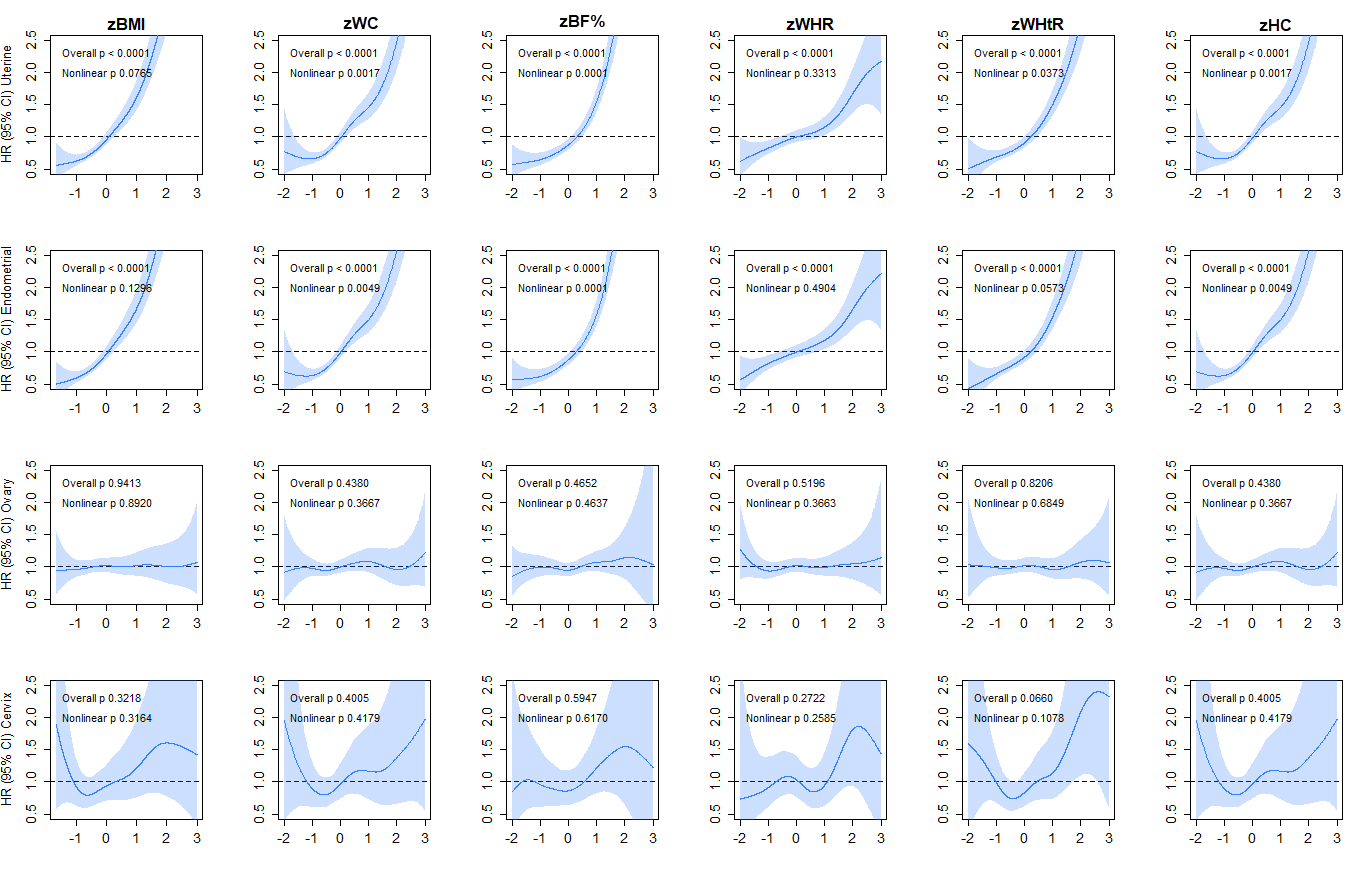
**

**Figure S8.** Association of adiposity markers with uterine, endometrial, ovary and cervical cancer incidence adjusted for sex-related covariates.

Penalised splines were used to present the association between adiposity markers and cancer outcomes. The adiposity markers were sex-standardised to 1-SD increment. Analyses were adjusted age, ethnicity, education, deprivation, smoking, dietary intake (alcohol, fruits & vegetables, red & processed meat, and oily fish), discretionary sedentary behaviour, physical activity, age menarche, hormonal replacement, age first and las live birth. BMI: Body Mass Index, BF%: Body Fat Percentage, WHR: waits hip ratio, WHTR: Waist height ratio, HC: hip circumference, HR: Hazard Ratio. Shaded areas represent 95% confidence intervals. Stomach no cardia did not have enough cases to perform the analyses for mortality (n>5). P-value for linear association corrected for multiple testing (Padj), p-value for non-linear association corrected for multiple testing (P-nlinear). Participants classified as underweight (BMI < 18.5 kg/m2 were excluded from the analyses (n = 2629).


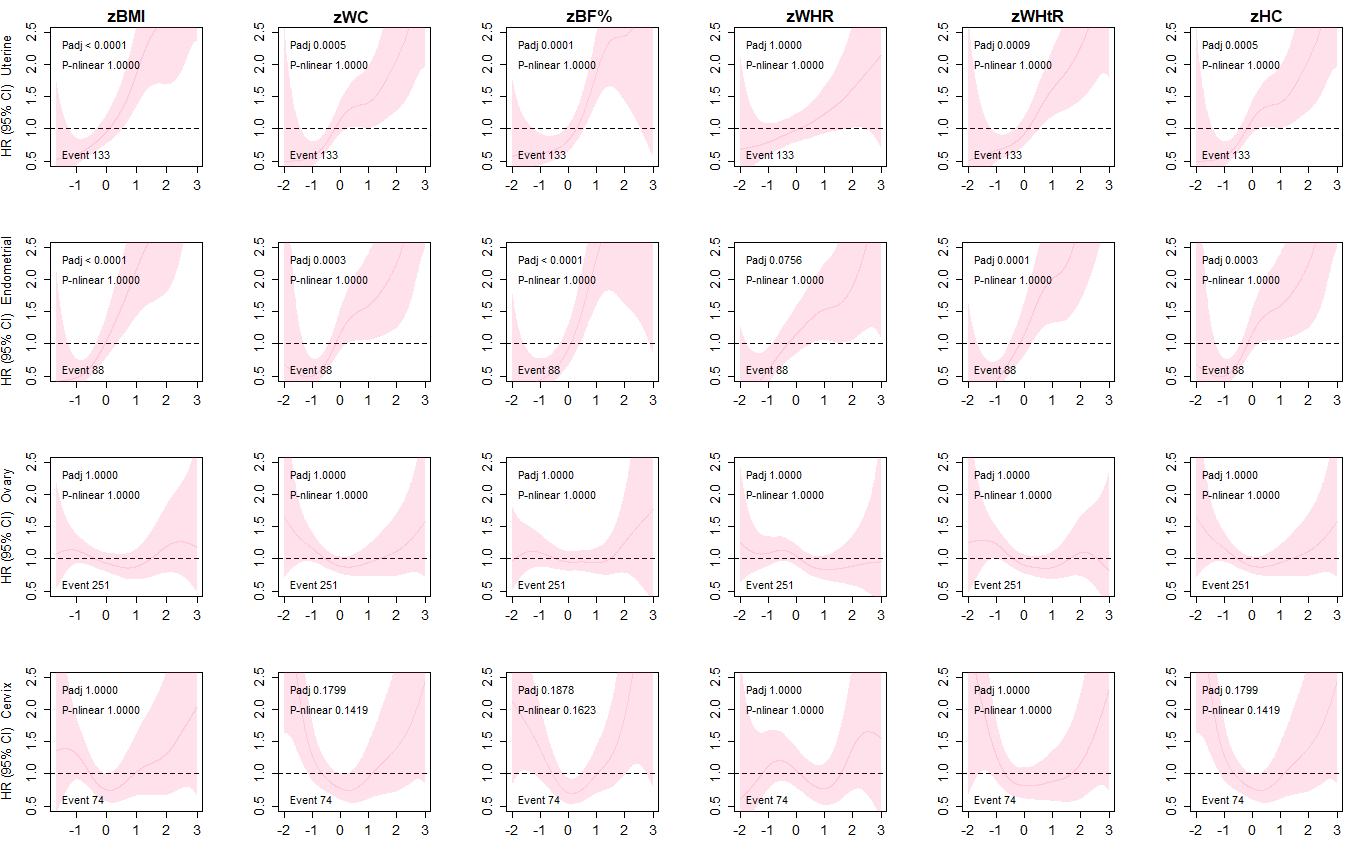


**Figure S9.** Association of adiposity markers with uterine, endometrial, ovary and cervical cancer mortality.

Penalised splines were used to present the association between adiposity markers and cancer outcomes. The adiposity markers were sex-standardised to 1-SD increment. Analyses were adjusted age, ethnicity, education, deprivation, smoking, dietary intake (alcohol, fruits & vegetables, red & processed meat, and oily fish), discretionary sedentary behaviour and physical activity. BMI: Body Mass Index, BF%: Body Fat Percentage, WHR: waits hip ratio, WHTR: Waist height ratio, HC: hip circumference, HR: Hazard Ratio. Shaded areas represent 95% confidence intervals. P-value for linear association corrected for multiple testing (Padj), p-value for non-linear association corrected for multiple testing (P-nlinear). Participants classified as underweight (BMI < 18.5 kg/m2 were excluded from the analyses (n = 2629).


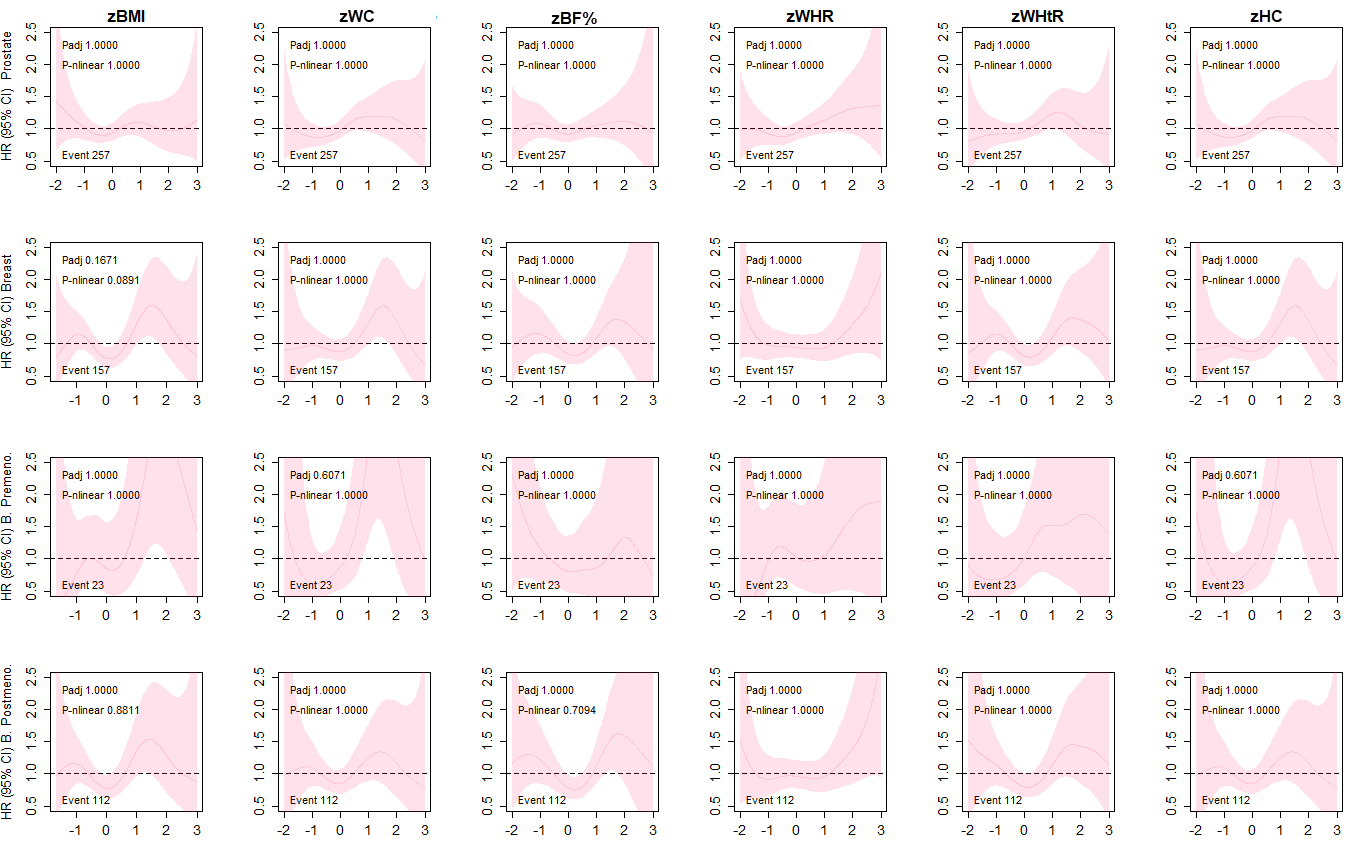


**Figure S10.** Association of adiposity markers with prostate, testicular cancer in men and breast cancer in pre- and post-menopausal women mortality.

Penalised splines were used to present the association between adiposity markers and cancer outcomes. The adiposity markers were sex-standardised to 1-SD increment. Analyses were adjusted age, ethnicity, education, deprivation, smoking, dietary intake (alcohol, fruits & vegetables, red & processed meat, and oily fish), discretionary sedentary behaviour and physical activity. BMI: Body Mass Index, BF%: Body Fat Percentage, WHR: waits hip ratio, WHTR: Waist height ratio, HC: hip circumference, HR: Hazard Ratio. Shaded areas represent 95% confidence intervals. P-value for linear association corrected for multiple testing (Padj), p-value for non-linear association corrected for multiple testing (P-nlinear). Participants classified as underweight (BMI < 18.5 kg/m2 were excluded from the analyses (n = 2629).

**
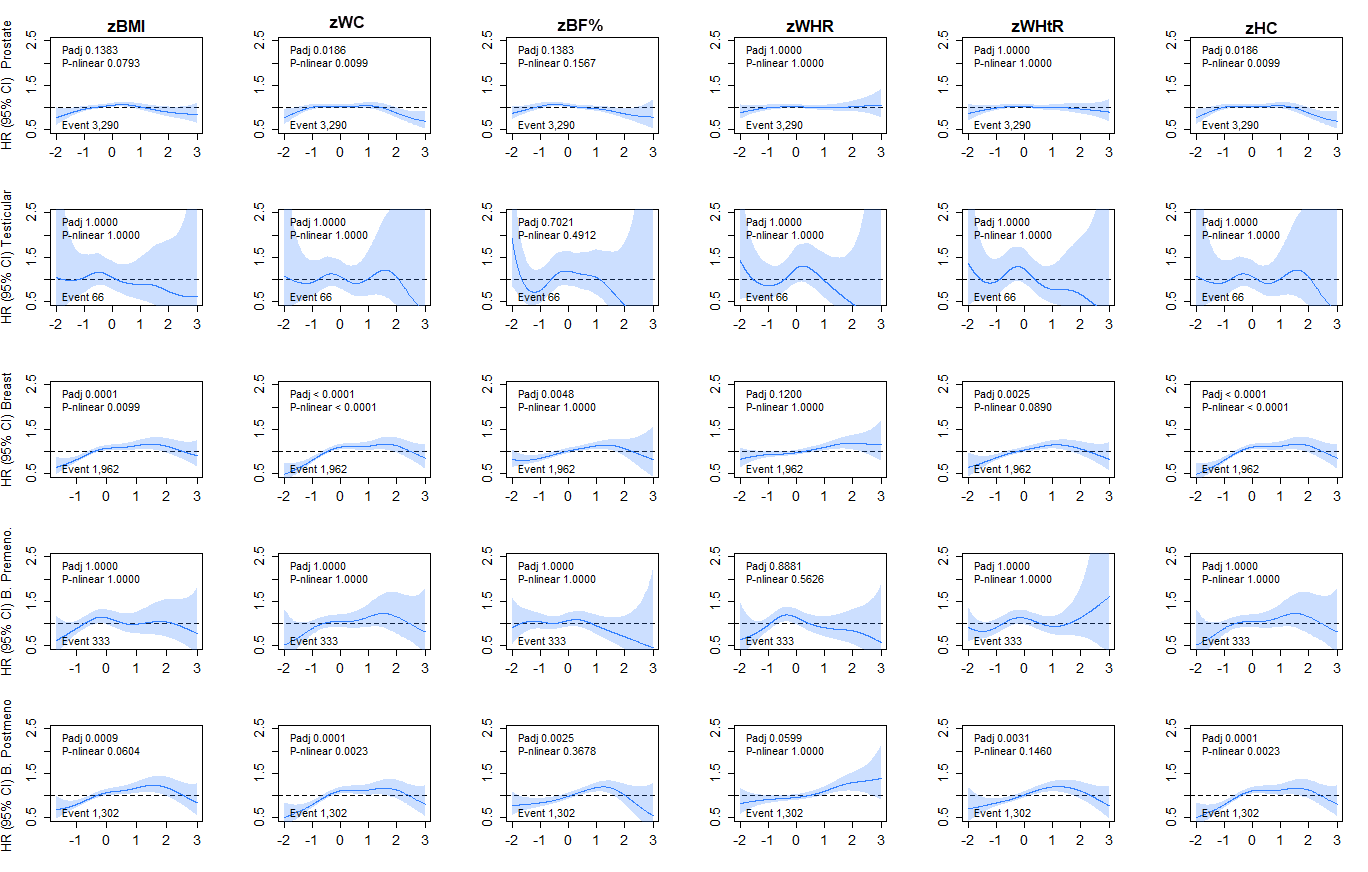
**

**Figure S11.** Association of adiposity markers with prostate, testicular, and breast cancer incidence additionally adjusted for sex-related covariates.

Penalised splines were used to present the association between adiposity markers and cancer outcomes. The adiposity markers were sex-standardised to 1-SD increment. Analyses were adjusted age, ethnicity, education, deprivation, smoking, dietary intake (alcohol, fruits & vegetables, red & processed meat, and oily fish), discretionary sedentary behaviour, physical activity, hormonal replacement, age menarche, age first and last live birth for breast and family history for prostate cancer. BMI: Body Mass Index, BF%: Body Fat Percentage, WHR: waits hip ratio, WHTR: Waist height ratio, HC: hip circumference, HR: Hazard Ratio. Shaded areas represent 95% confidence intervals. P-value for linear association corrected for multiple testing (Padj), p-value for non-linear association corrected for multiple testing (P-nlinear). Participants classified as underweight (BMI < 18.5 kg/m2 were excluded from the analyses (n = 2629).

**
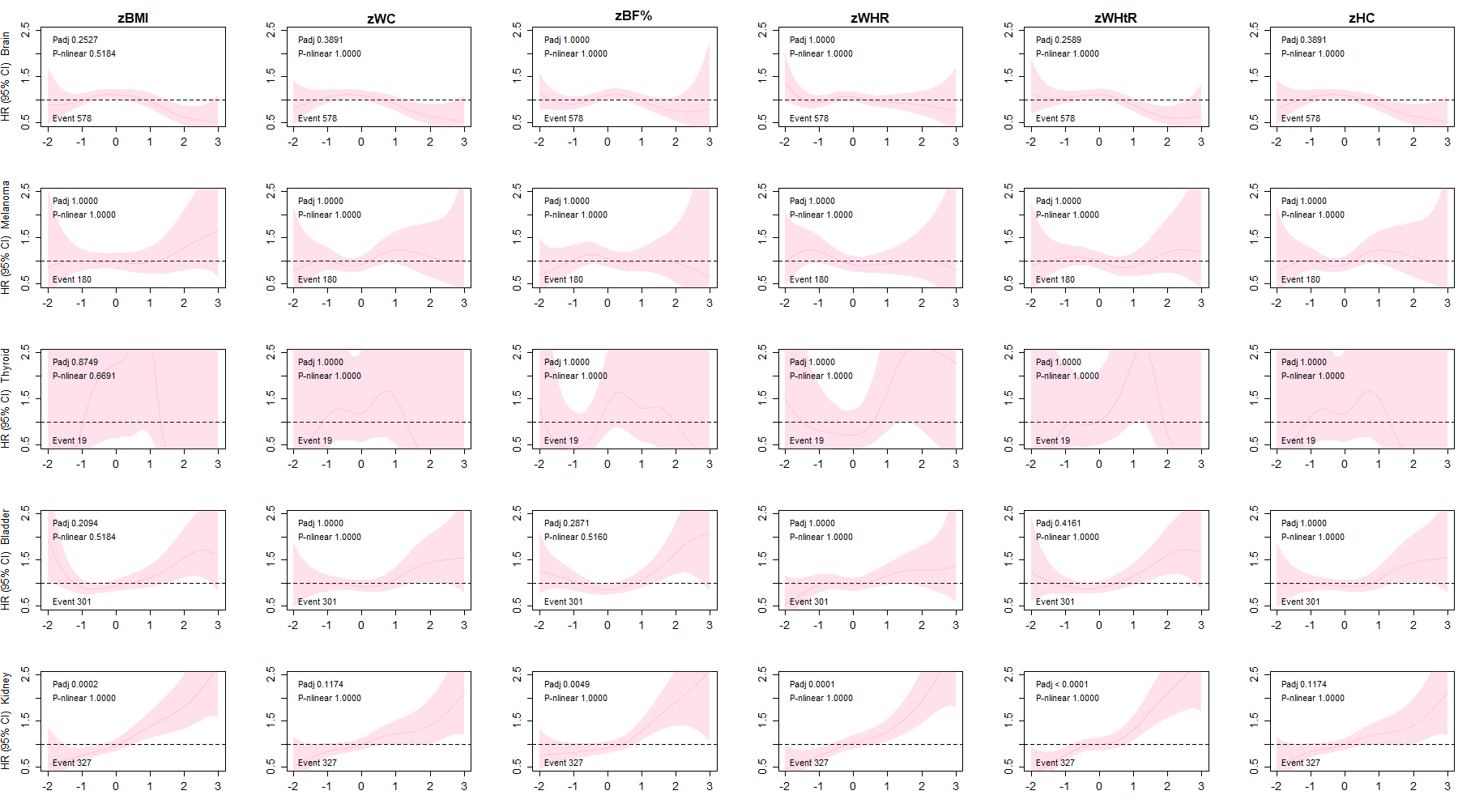
**

**Figure S12.** Association of adiposity markers with brain, melanoma, thyroid, bladder and kidney cancer mortality.

Penalised splines were used to present the association between adiposity markers and cancer outcomes. The adiposity markers were sex-standardised to 1-SD increment. Analyses were adjusted age, sex, ethnicity, education, deprivation, smoking, dietary intake (alcohol, fruits & vegetables, red & processed meat, and oily fish), discretionary sedentary behaviour and physical activity, melanoma cancer also was adjusted for sun exposition. BMI: Body Mass Index, BF%: Body Fat Percentage, WHR: waits hip ratio, WHTR: Waist height ratio, HC: hip circumference, HR: Hazard Ratio. Shaded areas represent 95% confidence intervals. P-value for linear association corrected for multiple testing (Padj), p-value for non-linear association corrected for multiple testing (P-nlinear). Participants classified as underweight (BMI < 18.5 kg/m2 were excluded from the analyses (n = 2629).


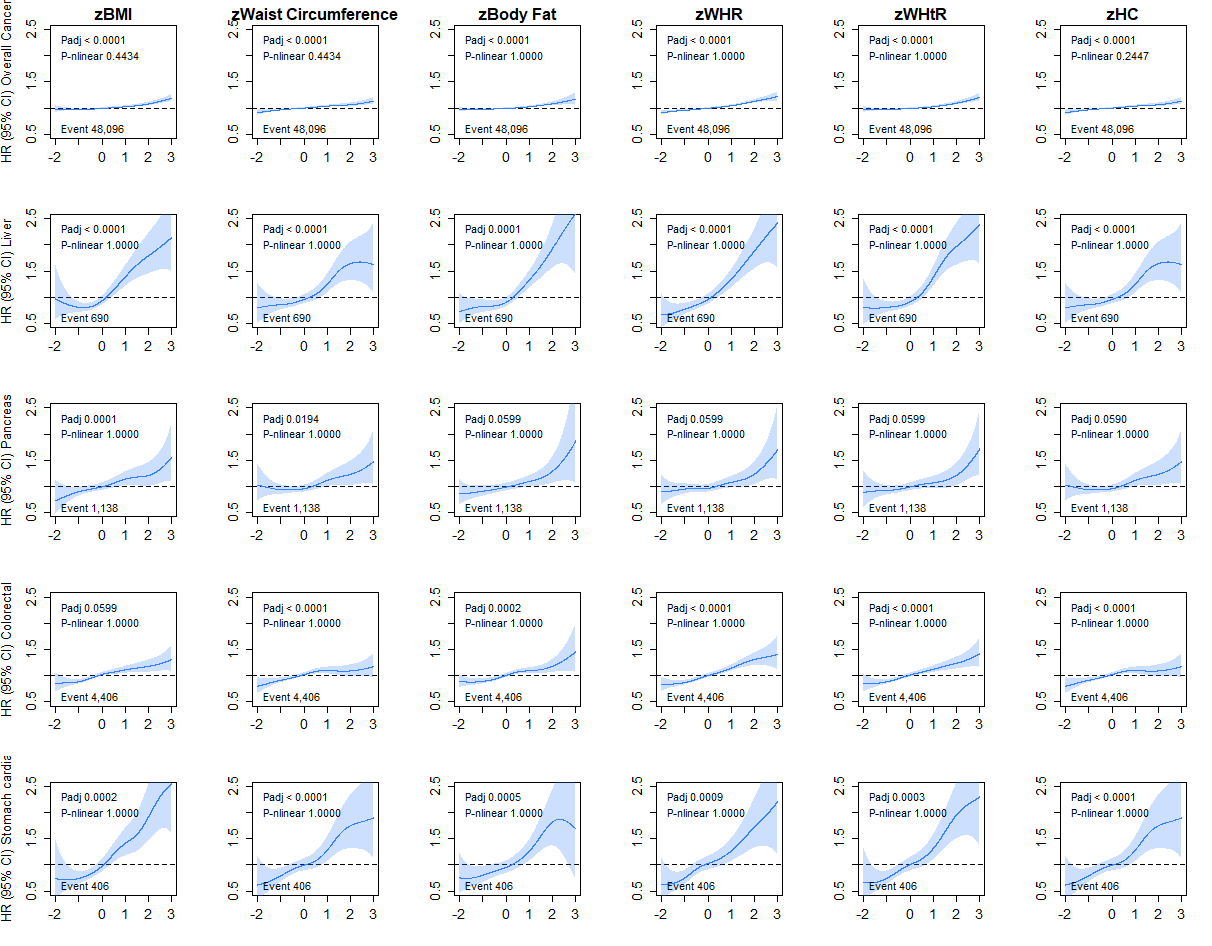


**Figure S13**. Association of Adiposity markers with overall, liver, pancreas, colorectal cancer and stomach cardia incidence with underweight people

Penalised splines were used to present the association between adiposity markers and cancer outcomes. The adiposity markers were sex-standardised to 1-SD increment. Analyses were adjusted age, sex, ethnicity, education, deprivation, smoking, dietary intake (alcohol, fruits & vegetables, red & processed meat, and oily fish), discretionary sedentary behaviour and physical activity. BMI: Body Mass Index, BF%: Body Fat Percentage, WHR: waits hip ratio, WHTR: Waist height ratio, HC: hip circumference, HR: Hazard Ratio. Shaded areas represent 95% confidence intervals. P-value for linear association corrected for multiple testing (Padj), p-value for non-linear association corrected for multiple testing (P-nlinear).


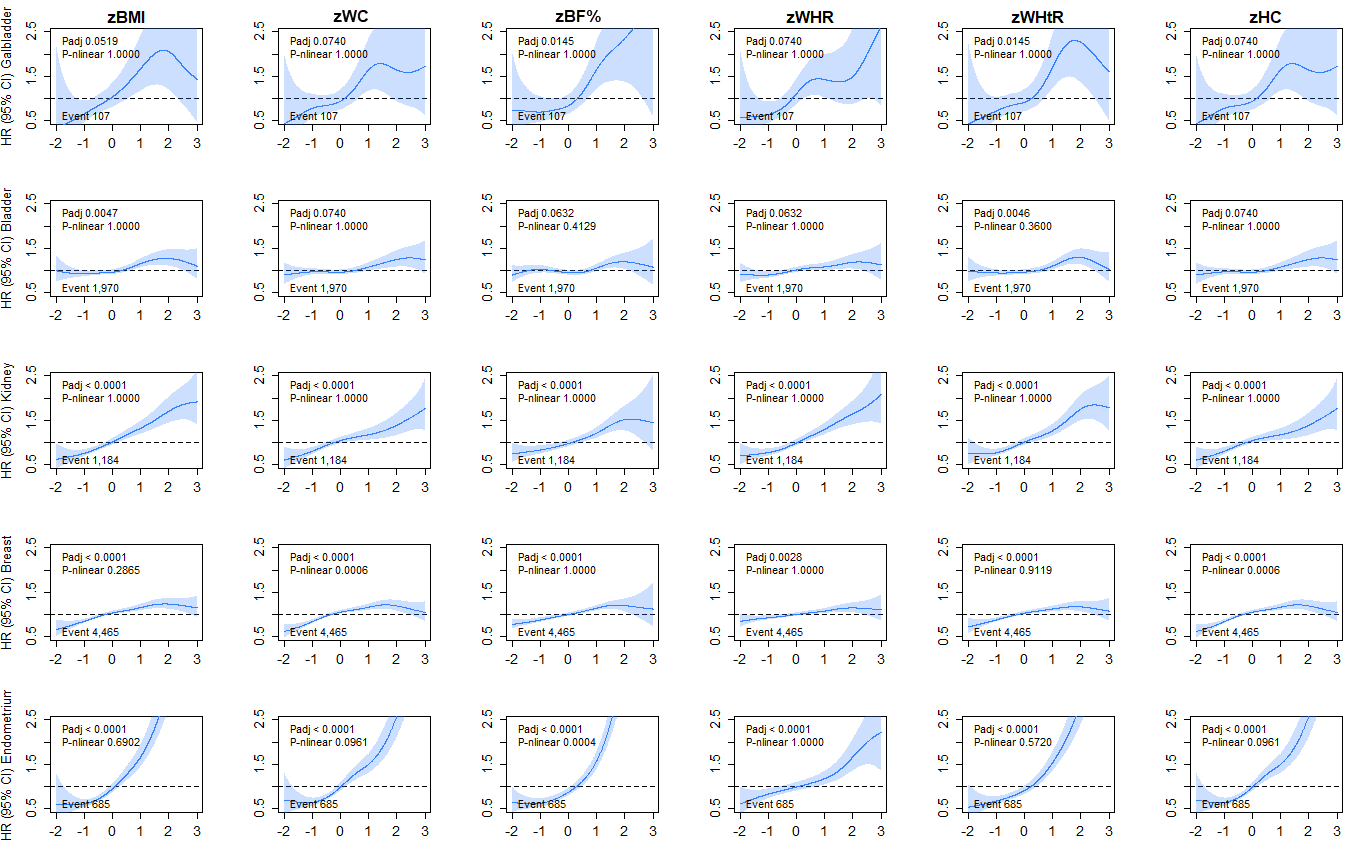


**Figure S14**. Association of Adiposity markers with gallbladder, bladder, kidney, breast and endometrium cancer incidence with underweight people

Penalised splines were used to present the association between adiposity markers and cancer outcomes. The adiposity markers were sex-standardised to 1-SD increment. Analyses were adjusted age, sex, ethnicity, education, deprivation, smoking, dietary intake (alcohol, fruits & vegetables, red & processed meat, and oily fish), discretionary sedentary behaviour and physical activity. BMI: Body Mass Index, BF%: Body Fat Percentage, WHR: waits hip ratio, WHTR: Waist height ratio, HC: hip circumference, HR: Hazard Ratio. Shaded areas represent 95% confidence intervals. P-value for linear association corrected for multiple testing (Padj), p-value for non-linear association corrected for multiple testing (P-nlinear).

**Table S1:** Association of adiposity markers with incidence from 24 cancer sites per 1 SD increase in adiposity markers.

| **Cancer site** | **Total/Event** | **BMI** | **WC** | **BF%** | **HC** | **WHR** | **WHTR** |
| --- | --- | --- | --- | --- | --- | --- | --- |
| Overall | 429,976/47,882 | **1.03 (1.02; 1.04)** | **1.04 (1.03; 1.05)** | **1.03 (1.02; 1.04)** | **1.04 (1.03; 1.05)** | **1.05 (1.04; 1.06)** | **1.04 (1.03; 1.05)** |
| Bladder | 437,087/1,961 | **1.09 (1.04; 1.14)** | 1.07 (1.02; 1.11) | 1.05 (1.00; 1.10) | 1.07 (1.02; 1.11) | **1.08 (1.03; 1.13)** | **1.08 (1.03; 1.13)** |
| Brain | 437,279/688 | 0.95 (0.88; 1.04) | 0.94 (0.87; 1.02) | 1.01 (0.93; 1.10) | 0.94 (0.87; 1.02) | 1.01 (0.93; 1.09) | 0.95 (0.87; 1.03) |
| Breast | 234,007/6,653 | **1.08 (1.05; 1.11)** | **1.09 (1.06; 1.12)** | **1.10 (1.07; 1.13)** | **1.09 (1.06; 1.12)** | **1.07 (1.04; 1.09)** | **1.08 (1.05; 1.10)** |
| Breast Postmenopausal | 139,546/4,168 | **1.10 (1.07; 1.14)** | **1.11 (1.08; 1.15)** | **1.12 (1.08; 1.16)** | **1.11 (1.08; 1.15)** | **1.09 (1.05; 1.12)** | **1.10 (1.07; 1.14)** |
| Breast Premenopausal | 58,701/1,442 | 0.99 (0.94; 1.05) | 1.03 (0.97; 1.08) | 1.04 (0.99; 1.10) | 1.03 (0.97; 1.08) | 1.00 (0.94; 1.06) | 0.98 (0.92; 1.04) |
| Cervix | 235,241/105 | 1.09 (0.90; 1.32) | 1.09 (0.90; 1.31) | 1.06 (0.86; 1.30) | 1.09 (0.90; 1.31) | 1.02 (0.84; 1.25) | 1.09 (0.89; 1.33) |
| Colorectal | 436,640/4,394 | **1.10 (1.06; 1.13)** | **1.07 (1.04; 1.11)** | **1.09 (1.05; 1.13)** | **1.07 (1.04; 1.11)** | **1.13 (1.10; 1.17)** | **1.12 (1.08; 1.15)** |
| Colon | 436859/3121 | **1.12 (1.08; 1.16)** | **1.10 (1.06; 1.13)** | **1.10 (1.06; 1.15)** | **1.10 (1.06; 1.13)** | **1.14 (1.10; 1.18)** | **1.13 (1.09; 1.18)** |
| Distal | 437155/1236 | **1.13 (1.07; 1.20)** | **1.11 (1.05; 1.18)** | **1.13 (1.07; 1.21)** | **1.11 (1.05; 1.18)** | **1.15 (1.08; 1.22)** | **1.15 (1.09; 1.22)** |
| Proximal | 437154/1729 | **1.14 (1.09; 1.20)** | **1.12 (1.07; 1.17)** | **1.13 (1.07; 1.19)** | **1.12 (1.07; 1.17)** | **1.16 (1.10; 1.22)** | **1.16 (1.11; 1.22)** |
| Rectum | 437092/1962 | **1.07 (1.02; 1.12)** | 1.04 (1.00; 1.09) | **1.07 (1.02; 1.13)** | 1.04 (1.00; 1.09) | **1.11 (1.06; 1.17)** | **1.09 (1.04; 1.14)** |
| Endometrium | 235,095/1,068 | **1.73 (1.65; 1.82)** | **1.63 (1.55; 1.71)** | **1.78 (1.66; 1.91)** | **1.63 (1.55; 1.71)** | **1.29 (1.22; 1.37)** | **1.69 (1.60; 1.79)** |
| Gallbladder | 437,380/107 | **1.33 (1.12; 1.58)** | **1.28 (1.08; 1.52)** | **1.50 (1.21; 1.86)** | **1.28 (1.08; 1.52)** | **1.32 (1.10; 1.59)** | **1.40 (1.16; 1.67)** |
| Kidney | 437,240/1,178 | **1.26 (1.20; 1.33)** | **1.18 (1.12; 1.25)** | **1.21 (1.13; 1.28)** | **1.18 (1.12; 1.25)** | **1.27 (1.20; 1.34)** | **1.26 (1.19; 1.33)** |
| Leukaemia | 437,276/1,129 | 1.07 (1.01; 1.14) | 1.08 (1.02; 1.15) | 1.02 (0.96; 1.09) | 1.08 (1.02; 1.15) | 1.07 (1.01; 1.14) | 1.05 (0.99; 1.12) |
| Liver | 437,322/688 | **1.27 (1.19; 1.36)** | **1.19 (1.11; 1.27)** | **1.32 (1.21; 1.43)** | **1.19 (1.11; 1.27)** | **1.32 (1.23; 1.42)** | **1.33 (1.23; 1.43)** |
| Lung | 241,636/509 | 0.99 (0.90; 1.09) | 0.92 (0.92; 1.11) | 1.00 (0.91; 1.10) | 0.91 (0.87; 0.95) | 1.07 (0.97; 1.17) | 1.01 (0.92; 1.12) |
| Lymphatic | 436,947/3,540 | **1.07 (1.04; 1.11)** | **1.08 (1.04; 1.12)** | 1.02 (0.99; 1.06) | **1.08 (1.04; 1.12)** | **1.06 (1.02; 1.10)** | 1.05 (1.01; 1.08) |
| Melanoma | 437,124/1,893 | 1.06 (1.01; 1.11) | **1.10 (1.05; 1.15)** | 1.03 (0.98; 1.08) | **1.10 (1.05; 1.15)** | 0.98 (0.93; 1.03) | 1.01 (0.96; 1.06) |
| Multiple Myeloma | 437,306/763 | 1.10 (1.02; 1.18) | 1.08 (1.01; 1.16) | 1.02 (0.95; 1.11) | 1.08 (1.01; 1.16) | 1.09 (1.01; 1.17) | 1.08 (1.00; 1.16) |
| Non-Hodgkin | 437,155/1,681 | 1.03 (0.98; 1.09) | 1.05 (1.00; 1.11) | 0.99 (0.94; 1.05) | 1.06 (1.00; 1.11) | 1.04 (0.99; 1.09) | 1.01 (0.96; 1.06) |
| Oesophagus | 241,662/297 | **1.23 (1.11; 1.38)** | **1.21 (1.09; 1.35)** | **1.19 (1.05; 1.35)** | **1.21 (1.09; 1.35)** | **1.18 (1.05; 1.32)** | **1.26 (1.13; 1.42)** |
| Oral | 241,661/290 | 0.96 (0.85; 1.09) | 0.99 (0.87; 1.11) | 0.90 (0.79; 1.02) | 0.99 (0.87; 1.11) | 0.95 (0.84; 1.08) | 0.97 (0.85; 1.10) |
| Ovary | 23,111/852 | 1.01 (0.93; 1.08) | 1.02 (0.95; 1.10) | 1.03 (0.96; 1.11) | 1.02 (0.95; 1.10) | 1.03 (0.96; 1.10) | 1.01 (0.94; 1.09) |
| Pancreas | 437,271/1,136 | **1.12 (1.06; 1.19)** | **1.09 (1.03; 1.16)** | **1.11 (1.04; 1.18)** | **1.09 (1.03; 1.16)** | 1.08 (1.02; 1.15) | **1.10 (1.03; 1.17)** |
| Prostate | 436,554/7,252 | **0.92 (0.90; 0.95)** | **0.94 (0.91; 0.96)** | **0.91 (0.89; 0.93)** | **0.94 (0.91; 0.96)** | **0.95 (0.92; 0.97)** | **0.92 (0.90; 0.95)** |
| Stomach | 437,294/747 | **1.24 (1.15; 1.32)** | **1.14 (1.07; 1.22)** | **1.16 (1.08; 1.26)** | **1.14 (1.07; 1.22)** | **1.21 (1.13; 1.30)** | **1.22 (1.13; 1.31)** |
| S. Cardia | 437338/404 | **1.35 (1.23; 1.47)** | **1.25 (1.14; 1.36)** | **1.27 (1.15; 1.42)** | **1.25 (1.14; 1.36)** | **1.29 (1.17; 1.42)** | **1.33 (1.21; 1.46)** |
| S. No Cardia | 437370/187 | 1.11 (0.97; 1.28) | 0.99 (0.86; 1.14) | 1.08 (0.93; 1.26) | 0.99 (0.86; 1.14) | **1.26 (1.09; 1.45**) | 1.15 (1.00; 1.33) |
| Testis | 437,379/67 | 0.86 (0.66; 1.13) | 0.93 (0.72; 1.20) | 0.84 (0.65; 1.08) | 0.93 (0.72; 1.20) | 0.90 (0.70; 1.17) | 0.83 (0.63; 1.09) |
| Thyroid | 437,340/284 | 1.11 (0.99; 1.25) | 1.13 (1.01; 1.27) | 1.11 (0.98; 1.26) | 1.13 (1.01; 1.27) | **1.23 (1.10; 1.38)** | 1.19 (1.05; 1.33) |
| Uterine | 235,061/1,188 | **1.68 (1.60; 1.75)** | **1.58 (1.51; 1.66)** | **1.70 (1.60; 1.82)** | **1.58 (1.51; 1.66)** | **1.26 (1.19; 1.34)** | **1.63 (1.55; 1.72)** |

_Data is presented as Hazar Ratio and their 95% confidence interval. Analyses were adjusted age, sex, ethnicity, education, deprivation, smoking, dietary intake (alcohol, fruits & vegetables, red & processed meat, and oily fish), discretionary sedentary behaviour and physical activity. BMI: Body Mass Index, BF%: Body Fat Percentage, WHR: waits hip ratio, WHTR: Waist height ratio, HC: hip circumference. P-values are corrected for multiple testing by using the Holm’s method. In_ **_bold_** _are those associations statistically significant after correcting for multiple testing._

**Table S2:** Association of adiposity markers with mortality from 24 cancer sites per 1 SD increase in adiposity markers.

| **Cancer site** | **Total/Event** | **BMI** | **WC** | **BF%** | **HC** | **WHR** | **WHTR** |
| --- | --- | --- | --- | --- | --- | --- | --- |
| Overall | 435,378/11,265 | **1.05 (1.03; 1.07)** | **1.03 (1.02; 1.07)** | **1.06 (1.04; 1.08)** | **1.04 (1.02; 1.06)** | **1.08 (1.06; 1.10)** | **1.08 (1.06; 1.10)** |
| Bladder | 437,383/301 | 1.11 (0.99; 1.24) | 1.10 (0.98; 1.22) | 1.09 (0.97; 1.24) | 1.10 (0.98; 1.22) | 1.13 (1.01; 1.27) | 1.16 (1.03; 1.30) |
| Brain | 437,339/578 | 0.92 (0.84; 1.01) | 0.91 (0.83; 0.99) | 0.98 (0.90; 1.07) | 0.91 (0.83; 0.99) | 0.95 (0.87; 1.04) | 0.89 (0.82; 0.98) |
| Breast | 235,252/477 | **1.12 (1.02; 1.23)** | **1.13 (1.03; 1.23)** | **1.17 (1.06; 1.30)** | **1.13 (1.03; 1.23)** | 1.09 (1.00; 1.20) | **1.11 (1.01; 1.22)** |
| Breast Postmenopausal | 140,365/307 | 1.14 (1.01; 1.28) | 1.13 (1.01; 1.26) | 1.19 (1.05; 1.36) | 1.13 (1.01; 1.26) | 1.15 (1.03; 1.29) | 1.15 (1.02; 1.29) |
| Breast Premenopausal | 58,936/89 | 1.16 (0.96; 1.41) | 1.19 (0.99; 1.45) | 1.23 (1.00; 1.52) | 1.19 (0.99; 1.45) | 0.98 (0.78; 1.23) | 1.06 (0.86; 1.32) |
| Cervix | 235,263/21 | 1.24 (0.83; 1.85) | 1.12 (0.74; 1.69) | 1.36 (0.85; 2.19) | 1.12 (0.74; 1.69) | 1.00 (0.64; 1.55) | 1.10 (0.71; 1.70) |
| Colorectal | 437,336/1,151 | **1.11 (1.04; 1.17)** | 1.08 (1.02; 1.15) | 1.10 (1.03; 1.17) | 1.08 (1.02; 1.15) | **1.14 (1.08; 1.21)** | **1.13 (1.06; 1.20)** |
| Colon | 437355/655 | 1.11 (1.02; 1.20) | **1.13 (1.04; 1.22)** | 1.08 (0.99; 1.18) | **1.13 (1.04; 1.22)** | 1.12 (1.04; 1.22) | 1.12 (1.04; 1.22) |
| Proximal | 437390/156 | 1.16 (0.99; 1.36) | 1.14 (0.98; 1.34) | 1.16 (0.97; 1.38) | 1.14 (0.98; 1.34) | 1.26 (1.08; 1.47) | 1.22 (1.04; 1.43) |
| Rectum | 437374/498 | 1.10 (1.01; 1.21) | 1.02 (0.93; 1.12) | 1.13 (1.03; 1.24) | 1.02 (0.93; 1.12) | **1.17 (1.07; 1.28)** | 1.14 (1.04; 1.25) |
| Endometrium | 235,263/128 | **1.70 (1.47; 1.97)** | **1.56 (1.34; 1.81)** | **1.84 (1.50; 2.26)** | **1.56 (1.34; 1.81)** | **1.35 (1.14; 1.60)** | **1.67 (1.42; 1.97)** |
| Gallbladder | 437,389/57 | 1.38 (1.09; 1.75) | 1.30 (1.03; 1.64) | **1.61 (1.20; 2.16)** | 1.30 (1.03; 1.64) | 1.41 (1.11; 1.80) | **1.50 (1.18; 1.92)** |
| Kidney | 437,371/327 | **1.32 (1.19; 1.46)** | **1.21 (1.09; 1.34)** | **1.30 (1.15; 1.46)** | **1.21 (1.09; 1.34)** | **1.36 (1.23; 1.50)** | **1.37 (1.24; 1.53)** |
| Leukaemia | 437,367/405 | 1.10 (0.99; 1.22) | 1.12 (1.02; 1.23) | 1.05 (0.95; 1.17) | 1.12 (1.02; 1.23) | 1.01 (0.91; 1.12) | 1.05 (0.95; 1.16) |
| Liver | 437,361/434 | **1.31 (1.21; 1.43)** | **1.25 (1.15; 1.36)** | **1.37 (1.23; 1.51)** | **1.25 (1.15; 1.36)** | **1.31 (1.20; 1.43)** | **1.36 (1.24; 1.49)** |
| Lung | 241,684/246 | 0.996(0.83; 1.10) | 1.00 (0.87; 1.15) | 1.00 (0.91; 1.10) | 0.91 (0.80; 1.05) | 1.02 (0.90; 1.17) | 0.98 (0.85; 1.13) |
| Lymphatic | 437,333/1,063 | **1.10 (1.04; 1.18)** | **1.10 (1.04; 1.17)** | 1.03 (0.96; 1.10) | **1.10 (1.04; 1.17)** | 1.08 (1.01; 1.15) | 1.07 (1.01; 1.15) |
| Melanoma | 437,387/180 | 1.06 (0.90; 1.24) | 1.07 (0.92; 1.25) | 1.02 (0.87; 1.19) | 1.07 (0.92; 1.25) | 0.92 (0.79; 1.08) | 0.99 (0.84; 1.16) |
| Multiple Myeloma | 437,383/221 | 1.13 (0.98; 1.30) | 1.21 (1.06; 1.37) | 1.03 (0.89; 1.19) | 1.21 (1.06; 1.37) | 1.00 (0.87; 1.15) | 1.08 (0.94; 1.24) |
| Non-Hodgkin | 437,372/416 | 1.07 (0.96; 1.18) | 1.01 (0.91; 1.11) | 0.98 (0.88; 1.09) | 1.01 (0.91; 1.11) | **1.18 (1.07; 1.30)** | 1.07 (0.97; 1.19) |
| Oesophagus | 241,696/152 | **1.35 (1.16; 1.56)** | 1.35 (1.18; 1.55) | **1.31 (1.10; 1.55)** | 1.35 (1.18; 1.55) | **1.22 (1.04; 1.43)** | **1.40 (1.20; 1.63)** |
| Oral | 241,700/45 | 0.86 (0.63; 1.18) | 0.82 (0.60; 1.12) | 0.94 (0.69; 1.27) | 0.82 (0.60; 1.12) | 1.06 (0.78; 1.42) | 0.89 (0.65; 1.21) |
| Ovary | 235,252/390 | 1.02 (0.91; 1.13) | 1.03 (0.93; 1.15) | 1.03 (0.93; 1.16) | 1.03 (0.93; 1.15) | 0.95 (0.86; 1.06) | 0.98 (0.88; 1.09) |
| Pancreas | 437,320/911 | **1.13 (1.06; 1.21)** | 1.10 (1.03; 1.17) | 1.11 (1.03; 1.19) | 1.10 (1.03; 1.17) | 1.11 (1.03; 1.18) | **1.11 (1.04; 1.19)** |
| Prostate | 202,112/632 | 1.04 (0.96; 1.13) | 1.04 (0.96; 1.12) | 1.01 (0.93; 1.10) | 1.04 (0.96; 1.12) | 1.11 (1.02; 1.21) | 1.07 (0.98; 1.16) |
| Stomach | 437,368/318 | **1.22 (1.10; 1.35)** | 1.12 (1.01; 1.24) | 1.09 (0.97; 1.22) | 1.12 (1.01; 1.24) | **1.20 (1.07; 1.34)** | **1.19 (1.06; 1.33)** |
| S. Cardia | 437390/58 | 1.55 (1.26; 1.92) | 1.40 (1.14; 1.73) | 1.47 (1.11; 1.94) | 1.40 (1.14; 1.73) | 1.53 (1.29; 1.80) | 1.56 (1.23; 1.97) |
| Thyroid | 437,390/19 | 1.11 (0.58; 2.12) | 0.77 (0.37; 1.60) | 1.13 (0.54; 2.36) | 0.77 (0.37; 1.60) | 2.01 (1.16; 3.48) | 1.51 (0.81; 2.80) |
| Uterine | 235,260/193 | **1.54 (1.36; 1.75)** | **1.45 (1.28; 1.64)** | **1.59 (1.35; 1.87)** | **1.45 (1.28; 1.64)** | **1.22 (1.06; 1.41)** | **1.48 (1.29; 1.70)** |

_Data is presented as Hazar Ratio and their 95% confidence interval. Analyses were adjusted age, sex, ethnicity, education, deprivation, smoking, dietary intake (alcohol, fruits & vegetables, red & processed meat, and oily fish), discretionary sedentary behaviour and physical activity. BMI: Body Mass Index, BF%: Body Fat Percentage, WHR: waits hip ratio, WHTR: Waist height ratio, HC: hip circumference. P-values are corrected for multiple testing by using the Holm’s method. In_ **_bold_** _are those associations statistically significant after correcting for multiple testing._

**Table S3:** C-Index for the predictive ability of BMI versus other adiposity markers.

|  | **BMI** | **Fat %** | | **WC** | |
| --- | --- | --- | --- | --- | --- |
|  | **C (95% CI)** | **ΔC (95% CI)** | **P** | **ΔC (95% CI)** | **P** |
| **Incidence** |  |  |  |  |  |
| Overall | 0.6546 (0.6521 to 0.6570) | -0.0003 (-0.0038 to 0.0031) | 0.85 | <0.0001 (-0.0035 to 0.0035) | 0.99 |
| Bladder | 0.7764 (0.7658 to 0.7871) | -0.0005 (-0.0157 to 0.0146) | 0.95 | -0.0006 (-0.0158 to 0.0145) | 0.93 |
| Brain | 0.6277 (0.6078 to 0.6477) | -0.0008 (-0.0290 to 0.0274) | 0.96 | -0.0005 (-0.0288 to 0.0277) | 0.97 |
| Colorectal | 0.6789 (0.6695 to 0.6882) | -0.0016 (-0.0148 to 0.0116) | 0.81 | -0.0003 (-0.0135 to 0.0129) | 0.96 |
| Gall bladder | 0.7313 (0.6822 to 0.7805) | -0.0041 (-0.0737 to 0.0655) | 0.91 | 0.0006 (-0.0689 to 0.0701) | 0.99 |
| Kidney | 0.7171 (0.7021 to 0.7322) | -0.0063 (-0.0276 to 0.0150) | 0.56 | -0.0042 (-0.0255 to 0.0171) | 0.70 |
| Leukaemia | 0.6924 (0.6769 to 0.7080) | -0.0013 (-0.0233 to 0.0207) | 0.90 | 0.0001 (-0.0218 to 0.0221) | 0.99 |
| Liver | 0.7270 (0.7071 to 0.7469) | -0.0006 (-0.0288 to 0.0275) | 0.96 | -0.0020 (-0.0302 to 0.0261) | 0.89 |
| Lung | 0.8210 (0.8130 to 0.8289) | -0.0008 (-0.0121 to 0.0104) | 0.88 | 0.0003 (-0.0110 to 0.0116) | 0.96 |
| Lymphoma | 0.6712 (0.6623 to 0.6801) | -0.0005 (-0.0131 to 0.0121) | 0.94 | 0.0004 (-0.0122 to 0.0130) | 0.95 |
| Melanoma | 0.6239 (0.6118 to 0.6359) | <0.0001 (-0.0171 to 0.0170) | 1.00 | 0.0014 (-0.0156 to 0.0185) | 0.87 |
| Multiple myeloma | 0.6797 (0.6604 to 0.6989) | 0.0007 (-0.0266 to 0.0279) | 0.96 | -0.0002 (-0.0274 to 0.0270) | 0.99 |
| Non-Hodgkin lymphoma | 0.6637 (0.6508 to 0.6766) | -0.0001 (-0.0183 to 0.0181) | 0.99 | 0.0006 (-0.0176 to 0.0188) | 0.95 |
| Oesophageal | 0.7703 (0.7545 to 0.7862) | <0.0001 (-0.0224 to 0.0225) | 1.00 | -0.0010 (-0.0234 to 0.0214) | 0.93 |
| Oral | 0.6761 (0.6583 to 0.6940) | 0.0011 (-0.0241 to 0.0264) | 0.93 | 0.0002 (-0.0250 to 0.0255) | 0.99 |
| Pancreatic | 0.7006 (0.6849 to 0.7163) | -0.0022 (-0.0244 to 0.0200) | 0.84 | -0.0002 (-0.0224 to 0.0220) | 0.99 |
| Stomach | 0.7474 (0.7291 to 0.7657) | -0.0049 (-0.0309 to 0.0211) | 0.71 | -0.0021 (-0.0280 to 0.0239) | 0.88 |
| Thyroid | 0.6389 (0.6083 to 0.6694) | 0.0005 (-0.0427 to 0.0437) | 0.98 | 0.0009 (-0.0423 to 0.0441) | 0.97 |
| Breast | 0.5565 (0.5500 to 0.5629) | 0.0003 (-0.0088 to 0.0095) | 0.94 | 0.0009 (-0.0083 to 0.0100) | 0.85 |
| Cervix | 0.5933 (0.5458 to 0.6408) | -0.0025 (-0.0697 to 0.0647) | 0.94 | -0.0011 (-0.0683 to 0.0661) | 0.97 |
| Endometrium | 0.6974 (0.6815 to 0.7132) | -0.0135 (-0.0359 to 0.0090) | 0.24 | -0.0114 (-0.0339 to 0.0111) | 0.32 |
| Ovary | 0.6213 (0.6036 to 0.6389) | 0.0013 (-0.0237 to 0.0262) | 0.92 | 0.0005 (-0.0244 to 0.0255) | 0.97 |
| Uterine | 0.6801 (0.6651 to 0.6951) | -0.0127 (-0.0340 to 0.0085) | 0.24 | -0.0099 (-0.0312 to 0.0113) | 0.36 |
| Prostate | 0.6866 (0.6802 to 0.6929) | 0.0013 (-0.0077 to 0.0102) | 0.78 | -0.0003 (-0.0092 to 0.0087) | 0.95 |
| Testis | 0.6376 (0.5746 to 0.7007) | -0.0013 (-0.0904 to 0.0879) | 0.98 | -0.0008 (-0.0899 to 0.0884) | 0.99 |
| **Mortality** |  |  |  |  |  |
| Overall | 0.7269 (0.7219 to 0.7320) | -0.0012 (-0.0083 to 0.0059) | 0.74 | <0.0001 (-0.0072 to 0.0071) | 0.99 |
| Bladder | 0.8150 (0.7871 to 0.8428) | -0.0027 (-0.0422 to 0.0368) | 0.89 | -0.0001 (-0.0395 to 0.0393) | 1.00 |
| Brain | 0.6544 (0.6316 to 0.6773) | -0.0023 (-0.0346 to 0.0300) | 0.89 | -0.0002 (-0.0325 to 0.0320) | 0.99 |
| Colorectal | 0.6916 (0.6702 to 0.7130) | -0.0013 (-0.0316 to 0.0290) | 0.93 | 0.0008 (-0.0295 to 0.0310) | 0.96 |
| Gallbladder | 0.7754 (0.7108 to 0.8400) | -0.0114 (-0.1034 to 0.0805) | 0.81 | <0.0001 (-0.0914 to 0.0914) | 1.00 |
| Kidney | 0.7694 (0.7413 to 0.7976) | -0.0030 (-0.0428 to 0.0369) | 0.88 | -0.0045 (-0.0444 to 0.0354) | 0.83 |
| Leukaemia | 0.7516 (0.7257 to 0.7775) | -0.0016 (-0.0382 to 0.0350) | 0.93 | 0.0005 (-0.0361 to 0.0371) | 0.98 |
| Liver | 0.7373 (0.7120 to 0.7625) | -0.0019 (-0.0377 to 0.0339) | 0.92 | -0.0009 (-0.0367 to 0.0349) | 0.96 |
| Lung | 0.8498 (0.8403 to 0.8593) | -0.0011 (-0.0145 to 0.0124) | 0.88 | 0.0001 (-0.0133 to 0.0136) | 0.98 |
| Lymphoma | 0.7445 (0.7284 to 0.7606) | -0.0010 (-0.0238 to 0.0218) | 0.93 | <0.0001 (-0.0228 to 0.0228) | 1.00 |
| Melanoma | 0.6848 (0.6431 to 0.7265) | -0.0012 (-0.0601 to 0.0578) | 0.97 | 0.0005 (-0.0585 to 0.0594) | 0.99 |
| Multiple myeloma | 0.7582 (0.7230 to 0.7933) | 0.0013 (-0.0484 to 0.0509) | 0.96 | 0.0026 (-0.0470 to 0.0523) | 0.92 |
| Non-Hodgkin lymphoma | 0.7381 (0.7120 to 0.7642) | -0.0018 (-0.0387 to 0.0351) | 0.92 | -0.0006 (-0.0375 to 0.0363) | 0.97 |
| Oesophageal | 0.7915 (0.7705 to 0.8125) | 0.0008 (-0.0289 to 0.0306) | 0.96 | -0.0013 (-0.0311 to 0.0285) | 0.93 |
| Oral | 0.7927 (0.7529 to 0.8325) | -0.0003 (-0.0566 to 0.0561) | 0.99 | <0.0001 (-0.0563 to 0.0563) | 1.00 |
| Pancreatic | 0.7085 (0.6907 to 0.7262) | -0.0024 (-0.0275 to 0.0227) | 0.85 | -0.0003 (-0.0254 to 0.0248) | 0.98 |
| Stomach | 0.7388 (0.7095 to 0.7682) | -0.0050 (-0.0466 to 0.0366) | 0.81 | -0.0028 (-0.0444 to 0.0387) | 0.89 |
| Thyroid | 0.7801 (0.6681 to 0.8922) | 0.0047 (-0.1533 to 0.1627) | 0.95 | -0.0004 (-0.1590 to 0.1581) | 1.00 |
| Breast | 0.5917 (0.5660 to 0.6174) | 0.0050 (-0.0314 to 0.0413) | 0.79 | 0.0022 (-0.0342 to 0.0385) | 0.91 |
| Cervix | 0.7750 (0.6622 to 0.8878) | 0.0095 (-0.1490 to 0.1681) | 0.91 | -0.0011 (-0.1606 to 0.1585) | 0.99 |
| Endometrium | 0.7922 (0.7476 to 0.8367) | -0.0043 (-0.0675 to 0.0589) | 0.89 | -0.0097 (-0.0731 to 0.0537) | 0.76 |
| Ovary | 0.6819 (0.6538 to 0.7100) | -0.0007 (-0.0404 to 0.0391) | 0.97 | -0.0001 (-0.0399 to 0.0396) | 1.00 |
| Uterine | 0.7587 (0.7212 to 0.7963) | -0.0019 (-0.0551 to 0.0513) | 0.94 | -0.0038 (-0.0570 to 0.0494) | 0.89 |
| Prostate | 0.7727 (0.7521 to 0.7932) | -0.0013 (-0.0304 to 0.0277) | 0.93 | <0.0001 (-0.0291 to 0.0290) | 1.00 |
| Testis | 0.9708 (0.8372 to 1.1045) | -0.0077 (-0.2082 to 0.1927) | 0.94 | -0.0083 (-0.2096 to 0.1930) | 0.94 |

ΔC (95% CI): Difference between C-indices with the model with BMI and their 95% confidence interval, P: p-value for ΔC

Table S3 continuation: C-Index for adiposity markers.

|  | **HC** | | **WHR** | | **WHtR** | |
| --- | --- | --- | --- | --- | --- | --- |
|  | **ΔC (95% CI)** | **P** | **ΔC (95% CI)** | **P** | **ΔC (95% CI)** | **P** |
| **Incidence** |  |  |  |  |  |  |
| Overall | <0.0001 (-0.0035 to 0.0035) | 0.99 | 0.0003 (-0.0032 to 0.0037) | 0.88 | <0.0001 (-0.0034 to 0.0035) | 0.99 |
| Bladder | -0.0006 (-0.0158 to 0.0145) | 0.93 | -0.0003 (-0.0154 to 0.0148) | 0.97 | 0.0001 (-0.0151 to 0.0152) | 0.99 |
| Brain | -0.0005 (-0.0288 to 0.0277) | 0.97 | -0.0008 (-0.0290 to 0.0275) | 0.96 | 0.0004 (-0.0278 to 0.0286) | 0.98 |
| Colorectal | -0.0003 (-0.0135 to 0.0129) | 0.96 | 0.0010 (-0.0122 to 0.0142) | 0.88 | 0.0006 (-0.0126 to 0.0138) | 0.93 |
| Gallbladder | 0.0006 (-0.0689 to 0.0701) | 0.99 | -0.0014 (-0.0710 to 0.0681) | 0.97 | 0.0023 (-0.0671 to 0.0718) | 0.95 |
| Kidney | -0.0042 (-0.0255 to 0.0171) | 0.70 | <0.0001 (-0.0213 to 0.0212) | 1.00 | -0.0009 (-0.0221 to 0.0204) | 0.94 |
| Leukaemia | 0.0001 (-0.0218 to 0.0221) | 0.99 | <0.0001 (-0.0220 to 0.0220) | 1.00 | -0.0004 (-0.0224 to 0.0216) | 0.97 |
| Liver | -0.0020 (-0.0302 to 0.0261) | 0.89 | 0.0039 (-0.0241 to 0.0320) | 0.78 | 0.0032 (-0.0249 to 0.0313) | 0.83 |
| Lung | 0.0003 (-0.0110 to 0.0116) | 0.96 | 0.0009 (-0.0103 to 0.0122) | 0.87 | -0.0001 (-0.0114 to 0.0111) | 0.98 |
| Lymphoma | 0.0004 (-0.0122 to 0.0130) | 0.95 | -0.0001 (-0.0127 to 0.0125) | 0.99 | -0.0003 (-0.0129 to 0.0123) | 0.96 |
| Melanoma | 0.0014 (-0.0156 to 0.0185) | 0.87 | -0.0003 (-0.0173 to 0.0168) | 0.98 | -0.0007 (-0.0177 to 0.0164) | 0.94 |
| Multiple myeloma | -0.0002 (-0.0274 to 0.0270) | 0.99 | -0.0003 (-0.0276 to 0.0269) | 0.98 | -0.0002 (-0.0274 to 0.0270) | 0.99 |
| Non-Hodgkin lymphoma | 0.0006 (-0.0176 to 0.0188) | 0.95 | 0.0003 (-0.0180 to 0.0185) | 0.98 | -0.0002 (-0.0184 to 0.0180) | 0.98 |
| Oesophageal | -0.0010 (-0.0234 to 0.0214) | 0.93 | 0.0029 (-0.0195 to 0.0252) | 0.80 | 0.0024 (-0.0200 to 0.0248) | 0.83 |
| Oral | 0.0002 (-0.0250 to 0.0255) | 0.99 | -0.0004 (-0.0256 to 0.0249) | 0.98 | <0.0001 (-0.0253 to 0.0252) | 1.00 |
| Pancreatic | -0.0002 (-0.0224 to 0.0220) | 0.99 | 0.0006 (-0.0216 to 0.0228) | 0.96 | -0.0001 (-0.0223 to 0.0221) | 0.99 |
| Stomach | -0.0021 (-0.0280 to 0.0239) | 0.88 | -0.0006 (-0.0265 to 0.0254) | 0.97 | -0.0007 (-0.0266 to 0.0253) | 0.96 |
| Thyroid | 0.0009 (-0.0423 to 0.0441) | 0.97 | 0.0020 (-0.0412 to 0.0452) | 0.93 | 0.0017 (-0.0415 to 0.0449) | 0.94 |
| Breast | 0.0009 (-0.0083 to 0.0100) | 0.85 | -0.0026 (-0.0118 to 0.0065) | 0.58 | -0.0016 (-0.0107 to 0.0076) | 0.73 |
| Cervix | -0.0011 (-0.0683 to 0.0661) | 0.97 | -0.0006 (-0.0678 to 0.0665) | 0.98 | 0.0009 (-0.0663 to 0.0681) | 0.98 |
| Endometrium | -0.0114 (-0.0339 to 0.0111) | 0.32 | **-0.0425 (-0.0651 to -0.0199)** | **0.0002** | -0.0080 (-0.0304 to 0.0145) | 0.49 |
| Ovary | 0.0005 (-0.0244 to 0.0255) | 0.97 | 0.0004 (-0.0246 to 0.0254) | 0.98 | <0.0001 (-0.0250 to 0.0250) | 1.00 |
| Uterine | -0.0099 (-0.0312 to 0.0113) | 0.36 | **-0.0392 (-0.0605 to -0.0179)** | **0.0003** | -0.0076 (-0.0288 to 0.0136) | 0.48 |
| Prostate | -0.0003 (-0.0092 to 0.0087) | 0.95 | -0.0005 (-0.0095 to 0.0084) | 0.91 | <0.0001 (-0.0090 to 0.0089) | 0.99 |
| Testis | -0.0008 (-0.0899 to 0.0884) | 0.99 | -0.0004 (-0.0896 to 0.0887) | 0.99 | -0.0011 (-0.0902 to 0.0881) | 0.98 |
| **Mortality** |  |  |  |  |  |  |
| Overall | <0.0001 (-0.0072 to 0.0071) | 0.99 | 0.0012 (-0.0059 to 0.0083) | 0.75 | 0.0005 (-0.0067 to 0.0076) | 0.90 |
| Bladder | -0.0001 (-0.0395 to 0.0393) | 1.00 | 0.0001 (-0.0393 to 0.0395) | 1.00 | 0.0005 (-0.0389 to 0.0399) | 0.98 |
| Brain | -0.0002 (-0.0325 to 0.0320) | 0.99 | -0.0007 (-0.0330 to 0.0316) | 0.97 | 0.0011 (-0.0311 to 0.0334) | 0.95 |
| Colorectal | 0.0008 (-0.0295 to 0.0310) | 0.96 | 0.0013 (-0.0290 to 0.0315) | 0.93 | 0.0004 (-0.0299 to 0.0307) | 0.98 |
| Gall bladder | <0.0001 (-0.0914 to 0.0914) | 1.00 | 0.0010 (-0.0903 to 0.0923) | 0.98 | 0.0035 (-0.0877 to 0.0946) | 0.94 |
| Kidney | -0.0045 (-0.0444 to 0.0354) | 0.83 | 0.0016 (-0.0382 to 0.0414) | 0.94 | 0.0016 (-0.0381 to 0.0414) | 0.94 |
| Leukaemia | 0.0005 (-0.0361 to 0.0371) | 0.98 | -0.0013 (-0.0379 to 0.0354) | 0.95 | -0.0009 (-0.0375 to 0.0357) | 0.96 |
| Liver | -0.0009 (-0.0367 to 0.0349) | 0.96 | 0.0017 (-0.0340 to 0.0374) | 0.93 | 0.0019 (-0.0339 to 0.0376) | 0.92 |
| Lung | 0.0001 (-0.0133 to 0.0136) | 0.98 | 0.0004 (-0.0131 to 0.0138) | 0.96 | -0.0004 (-0.0139 to 0.0131) | 0.95 |
| Lymphoma | <0.0001 (-0.0228 to 0.0228) | 1.00 | -0.0004 (-0.0233 to 0.0224) | 0.97 | -0.0004 (-0.0232 to 0.0225) | 0.97 |
| Melanoma | 0.0005 (-0.0585 to 0.0594) | 0.99 | <0.0001 (-0.0590 to 0.0589) | 1.00 | -0.0015 (-0.0605 to 0.0575) | 0.96 |
| Multiple myeloma | 0.0026 (-0.0470 to 0.0523) | 0.92 | -0.0032 (-0.0530 to 0.0466) | 0.90 | -0.0011 (-0.0508 to 0.0487) | 0.97 |
| Non-Hodgkin lymphoma | -0.0006 (-0.0375 to 0.0363) | 0.97 | 0.0031 (-0.0337 to 0.0400) | 0.87 | 0.0005 (-0.0364 to 0.0374) | 0.98 |
| Oesophageal | -0.0013 (-0.0311 to 0.0285) | 0.93 | 0.0045 (-0.0251 to 0.0342) | 0.76 | 0.0036 (-0.0260 to 0.0333) | 0.81 |
| Oral | <0.0001 (-0.0563 to 0.0563) | 1.00 | 0.0014 (-0.0549 to 0.0576) | 0.96 | 0.0008 (-0.0555 to 0.0571) | 0.98 |
| Pancreatic | -0.0003 (-0.0254 to 0.0248) | 0.98 | 0.0008 (-0.0243 to 0.0259) | 0.95 | -0.0003 (-0.0254 to 0.0248) | 0.98 |
| Stomach | -0.0028 (-0.0444 to 0.0387) | 0.89 | -0.0007 (-0.0422 to 0.0408) | 0.97 | -0.0011 (-0.0426 to 0.0404) | 0.96 |
| Thyroid | -0.0004 (-0.1590 to 0.1581) | 1.00 | 0.0047 (-0.1532 to 0.1627) | 0.95 | 0.0035 (-0.1546 to 0.1616) | 0.97 |
| Breast | 0.0022 (-0.0342 to 0.0385) | 0.91 | -0.0023 (-0.0386 to 0.0341) | 0.90 | 0.0001 (-0.0363 to 0.0364) | 1.00 |
| Cervix | -0.0011 (-0.1606 to 0.1585) | 0.99 | -0.0018 (-0.1614 to 0.1579) | 0.98 | -0.0022 (-0.1619 to 0.1574) | 0.98 |
| Endometrium | -0.0097 (-0.0731 to 0.0537) | 0.76 | -0.0160 (-0.0796 to 0.0477) | 0.62 | -0.0020 (-0.0651 to 0.0611) | 0.95 |
| Ovary | -0.0001 (-0.0399 to 0.0396) | 1.00 | 0.0002 (-0.0396 to 0.0399) | 0.99 | -0.0001 (-0.0399 to 0.0396) | 0.99 |
| Uterine | -0.0038 (-0.0570 to 0.0494) | 0.89 | -0.0118 (-0.0653 to 0.0416) | 0.66 | -0.0028 (-0.0561 to 0.0504) | 0.92 |
| Prostate | <0.0001 (-0.0291 to 0.0290) | 1.00 | 0.0008 (-0.0283 to 0.0298) | 0.96 | 0.0003 (-0.0288 to 0.0293) | 0.98 |
| Testis | -0.0083 (-0.2096 to 0.1930) | 0.94 | 0.0197 (-0.1347 to 0.1741) | 0.80 | 0.0005 (-0.1879 to 0.1888) | 1.00 |

ΔC (95% CI): Difference between C-indices with the model with BMI and their 95% confidence interval, P: p-value for ΔC

**Table S4:** Association of adiposity markers with incidence from 24 cancer sites after accounting for competing risk.

| **Cancer site** | **Total/event** | **BMI** | **WC** | **BF%** | **WHR** | **WHTR** | **HC** |
| --- | --- | --- | --- | --- | --- | --- | --- |
| Overall | 425,604/43,458 | **1.08 (1.07; 1.09)** | **1.07 (1.06; 1.08)** | **1.06 (1.05; 1.07)** | **1.09 (1.08; 1.10)** | **1.09 (1.08; 1.10)** | **1.07 (1.06; 1.08)** |
| Bladder | 436,819/1,688 | **1.13 (1.07; 1.19)** | **1.10 (1.05; 1.15)** | **1.08 (1.03; 1.14)** | **1.13 (1.08; 1.19)** | **1.13 (1.08; 1.19)** | **1.10 (1.05; 1.15)** |
| Brain | 437,199/603 | 1.00 (0.91; 1.09) | 0.97 (0.89; 1.06) | 1.05 (0.96; 1.15) | 1.03 (0.95; 1.12) | 0.99 (0.91; 1.08) | 0.97 (0.89; 1.06) |
| Breast | 435,620/6,203 | **1.16 (1.13; 1.19)** | **1.16 (1.13; 1.19)** | **1.18 (1.15; 1.22)** | **1.14 (1.11; 1.17)** | **1.17 (1.14; 1.20)** | **1.16 (1.13; 1.19)** |
| Breast Postmenopausal | 139,183/3,804 | **1.16 (1.12; 1.20)** | **1.16 (1.12; 1.20)** | **1.16 (1.12; 1.20)** | **1.12 (1.08; 1.16)** | **1.16 (1.12; 1.20)** | **1.16 (1.12; 1.20)** |
| Breast Premenopausal | 58,656/1,396 | 1.02 (0.97; 1.08) | 1.06 (1.00; 1.12) | 1.07 (1.01; 1.13) | 1.02 (0.96; 1.08) | 1.01 (0.95; 1.07) | 1.06 (1.00; 1.12) |
| Cervix | 437,358/94 | 1.21 (0.99; 1.47) | 1.21 (1.00; 1.47) | 1.15 (0.92; 1.42) | 1.08 (0.88; 1.34) | 1.21 (0.98; 1.48) | 1.21 (1.00; 1.47) |
| Colorectal | 436,183/3,936 | **1.15 (1.11; 1.18)** | **1.11 (1.08; 1.15)** | **1.13 (1.09; 1.17)** | **1.19 (1.15; 1.23)** | **1.18 (1.14; 1.21)** | **1.11 (1.08; 1.15)** |
| Endometrium | 437,123/968 | **1.84 (1.75; 1.93)** | **1.71 (1.63; 1.79)** | **1.91 (1.78; 2.05)** | **1.37 (1.30; 1.45)** | **1.82 (1.72; 1.93)** | **1.71 (1.63; 1.79)** |
| Gallbladder | 437,364/91 | **1.46 (1.22; 1.76)** | **1.38 (1.15; 1.65)** | **1.61 (1.27; 2.03)** | **1.39 (1.15; 1.68)** | **1.55 (1.28; 1.89)** | **1.38 (1.15; 1.65)** |
| Kidney | 437,072/1,008 | **1.31 (1.23; 1.39)** | **1.21 (1.14; 1.28)** | **1.24 (1.16; 1.33)** | **1.33 (1.25; 1.41)** | **1.32 (1.24; 1.41)** | **1.21 (1.14; 1.28)** |
| Leukaemia | 437,120/964 | **1.15 (1.07; 1.22)** | **1.15 (1.08; 1.23)** | 1.07 (1.00; 1.14) | **1.14 (1.07; 1.22)** | **1.13 (1.05; 1.21)** | **1.15 (1.08; 1.23)** |
| Liver | 437,215/580 | **1.34 (1.24; 1.44)** | **1.23 (1.14; 1.33)** | **1.38 (1.26; 1.51)** | **1.39 (1.29; 1.49)** | **1.41 (1.30; 1.52)** | **1.23 (1.14; 1.33)** |
| Lung | 436,467/2,731 | 0.97 (0.93; 1.01) | 0.95 (0.92; 0.99) | 1.03 (0.99; 1.08) | **1.16 (1.11; 1.20)** | 1.05 (1.01; 1.09) | 0.95 (0.92; 0.99) |
| Lymphatic | 436,523/3,105 | **1.12 (1.08; 1.16)** | **1.12 (1.08; 1.17)** | 1.06 (1.02; 1.10) | **1.11 (1.07; 1.15)** | **1.10 (1.06; 1.15)** | **1.12 (1.08; 1.17)** |
| Melanoma | 436,931/1,698 | **1.08 (1.03; 1.14)** | **1.12 (1.06; 1.17)** | 1.04 (0.99; 1.10) | 0.99 (0.94; 1.05) | 1.03 (0.97; 1.08) | **1.12 (1.06; 1.17)** |
| Multiple Myeloma | 437,217/672 | **1.14 (1.05; 1.24)** | **1.13 (1.04; 1.22)** | 1.06 (0.97; 1.15) | **1.14 (1.05; 1.23)** | **1.14 (1.05; 1.24)** | **1.13 (1.04; 1.22)** |
| Non-Hodgkin | 436,948/1,473 | 1.07 (1.01; 1.13) | **1.08 (1.02; 1.14)** | 1.01 (0.96; 1.07) | 1.08 (1.02; 1.14) | 1.05 (1.00; 1.12) | **1.08 (1.02; 1.14)** |
| Oesophagus | 437,121/0,000 | **1.26 (1.18; 1.35)** | **1.17 (1.09; 1.25)** | **1.27 (1.18; 1.37)** | **1.36 (1.28; 1.45)** | **1.35 (1.26; 1.44)** | **1.17 (1.09; 1.25)** |
| Oral | 437,180/740 | 0.93 (0.86; 1.01) | 0.93 (0.86; 1.01) | 0.95 (0.88; 1.03) | 1.05 (0.97; 1.13) | 0.99 (0.91; 1.07) | 0.93 (0.86; 1.01) |
| Ovary | 437,172/787 | 1.08 (1.00; 1.16) | 1.08 (1.00; 1.16) | 1.09 (1.01; 1.18) | 1.10 (1.02; 1.18) | 1.09 (1.01; 1.17) | 1.08 (1.00; 1.16) |
| Pancreas | 437,125/990 | **1.19 (1.11; 1.27)** | **1.14 (1.07; 1.22)** | **1.16 (1.08; 1.24)** | **1.15 (1.08; 1.23)** | **1.18 (1.11; 1.26)** | **1.14 (1.07; 1.22)** |
| Prostate | 435,777/6,470 | 0.97 (0.94; 1.00) | 0.97 (0.95; 1.00) | **0.94 (0.92; 0.97)** | 0.99 (0.96; 1.01) | 0.98 (0.95; 1.00) | 0.97 (0.95; 1.00) |
| Stomach | 437,192/645 | **1.27 (1.17; 1.36)** | **1.15 (1.07; 1.24)** | **1.16 (1.07; 1.27)** | **1.27 (1.17; 1.37)** | **1.26 (1.17; 1.36)** | **1.15 (1.07; 1.24)** |
| Testis | 437,375/63 | 0.91 (0.69; 1.20) | 0.94 (0.72; 1.23) | 0.85 (0.65; 1.10) | 0.93 (0.71; 1.22) | 0.86 (0.65; 1.14) | 0.94 (0.72; 1.23) |
| Thyroid | 437,309/253 | 1.16 (1.02; 1.31) | **1.19 (1.06; 1.34)** | 1.13 (0.99; 1.29) | **1.21 (1.07; 1.37)** | **1.22 (1.08; 1.38)** | **1.19 (1.06; 1.34)** |
| Uterine | 437,077/1,076 | **1.79 (1.71; 1.88)** | **1.67 (1.59; 1.75)** | **1.83 (1.71; 1.95)** | **1.35 (1.28; 1.43)** | **1.77 (1.67; 1.86)** | **1.67 (1.59; 1.75)** |

_Data is presented as Hazar Ratio and their 95% confidence interval. Analyses were adjusted age, sex, ethnicity, education, deprivation, smoking, dietary intake (alcohol, fruits & vegetables, red & processed meat, and oily fish), discretionary sedentary behaviour and physical activity. BMI: Body Mass Index, BF%: Body Fat Percentage, WHR: waits hip ratio, WHTR: Waist height ratio, HC: hip circumference. A landmark analysis was performed to reduce the potential for reverse causality, with follow-up commencing two years after recruitment. P-values are corrected for multiple testing by using the Holm’s method. In_ **_bold_** _are those associations statistically significant after correcting for multiple testing. Participants classified as underweight (BMI < 18.5 kg/m2 were excluded from the analyses (n = 2629)._

**Table S5:** Association of adiposity markers with incidence from 24 cancer sites per 1 SD increase in adiposity markers with height as covariate.

| **Cancer site** | **Total/Event** | **BMI** | **WC** | **BF%** | **HC** | **WHR** | **WHTR** |
| --- | --- | --- | --- | --- | --- | --- | --- |
| Overall | 429,976/47,882 | **1.04 (1.03; 1.05)** | **1.02 (1.01; 1.03)** | **1.03 (1.02; 1.04)** | **1.02 (1.01; 1.03)** | **1.05 (1.04; 1.06)** | **1.05 (1.04; 1.06)** |
| Bladder | 437,087/1,961 | **1.09 (1.04; 1.14)** | 1.06 (1.01; 1.11) | 1.05 (1.00; 1.10) | 1.06 (1.01; 1.11) | **1.08 (1.03; 1.13)** | **1.09 (1.04; 1.14)** |
| Brain | 437,279/688 | 0.95 (0.88; 1.04) | 0.93 (0.85; 1.01) | 1.01 (0.93; 1.10) | 0.93 (0.85; 1.01) | 1.01 (0.93; 1.09) | 0.95 (0.88; 1.03) |
| Breast | 234,007/6,653 | **1.09 (1.06; 1.12)** | **1.08 (1.05; 1.10)** | **1.09 (1.06; 1.12)** | **1.08 (1.05; 1.10)** | **1.07 (1.05; 1.10)** | **1.10 (1.07; 1.13)** |
| Breast Postmenopausal | 139,546/4,168 | **1.11 (1.08; 1.15)** | **1.10 (1.06; 1.13)** | **1.11 (1.07; 1.15)** | **1.10 (1.06; 1.13)** | **1.09 (1.06; 1.13)** | **1.13 (1.09; 1.17)** |
| Breast Premenopausal | 58,700/1,442 | 1.00 (0.95; 1.06) | 1.00 (0.95; 1.06) | 1.04 (0.98; 1.09) | 1.00 (0.95; 1.06) | 1.00 (0.95; 1.06) | 1.00 (0.94; 1.06) |
| Cervix | 235,241/105 | 1.09 (0.90; 1.32) | 1.09 (0.90; 1.32) | 1.06 (0.86; 1.31) | 1.09 (0.90; 1.32) | 1.02 (0.84; 1.25) | 1.09 (0.89; 1.33) |
| Colorectal | 436,640/4,394 | **1.10 (1.07; 1.13)** | **1.07 (1.03; 1.10)** | **1.09 (1.06; 1.13)** | **1.07 (1.03; 1.10)** | **1.13 (1.10; 1.17)** | **1.13 (1.10; 1.17)** |
| Colon | 436,859/3,121 | **1.12 (1.08; 1.16)** | **1.08 (1.05; 1.12)** | **1.11 (1.06; 1.15)** | **1.08 (1.05; 1.12)** | **1.14 (1.10; 1.19)** | **1.15 (1.11; 1.19)** |
| Distal | 437,155/1,236 | **1.14 (1.07; 1.20)** | **1.11 (1.04; 1.17)** | **1.13 (1.07; 1.21)** | **1.11 (1.04; 1.17)** | **1.15 (1.09; 1.22)** | **1.17 (1.10; 1.24)** |
| Proximal | 437,154/1,729 | **1.15 (1.09; 1.20)** | **1.11 (1.06; 1.16)** | **1.13 (1.07; 1.19)** | **1.11 (1.06; 1.16)** | **1.16 (1.11; 1.22)** | **1.18 (1.12; 1.24)** |
| Rectum | 437,092/1,962 | **1.07 (1.02; 1.12)** | 1.03 (0.99; 1.08) | 1.07 (1.02; 1.13) | 1.03 (0.99; 1.08) | **1.12 (1.06; 1.17)** | **1.10 (1.05; 1.15)** |
| Endometrium | 235,095/1,068 | **1.74 (1.66; 1.82)** | **1.64 (1.56; 1.72)** | **1.78 (1.66; 1.91)** | **1.64 (1.56; 1.72)** | **1.29 (1.22; 1.37)** | **1.72 (1.63; 1.82)** |
| Gallbladder | 437,380/107 | **1.34 (1.13; 1.59)** | 1.28 (1.08; 1.52) | **1.50 (1.21; 1.86)** | 1.28 (1.08; 1.52) | **1.32 (1.10; 1.59)** | **1.42 (1.18; 1.71)** |
| Kidney | 437,240/1,178 | **1.27 (1.20; 1.34)** | **1.17 (1.10; 1.23)** | **1.21 (1.14; 1.29)** | **1.17 (1.10; 1.23)** | **1.27 (1.20; 1.35)** | **1.29 (1.22; 1.36)** |
| Leukaemia | 437,276/1,129 | 1.08 (1.02; 1.15) | 1.05 (0.99; 1.12) | 1.02 (0.96; 1.09) | 1.05 (0.99; 1.12) | 1.07 (1.01; 1.14) | 1.08 (1.01; 1.15) |
| Liver | 437,322/688 | **1.27 (1.19; 1.36)** | **1.19 (1.11; 1.28)** | **1.32 (1.21; 1.43)** | **1.19 (1.11; 1.28)** | **1.32 (1.23; 1.42)** | **1.34 (1.25; 1.44)** |
| Lung | 241,636/509 | 1.00 (0.90; 1.10) | 0.99 (0.90; 1.09) | 1.00 (0.91; 1.10) | 0.99 (0.90; 1.09) | 1.07 (0.98; 1.17) | 1.04 (0.94; 1.14) |
| Lymphatic | 436,947/3,540 | **1.08 (1.04; 1.12)** | 1.05 (1.01; 1.09) | 1.02 (0.99; 1.06) | 1.05 (1.01; 1.09) | **1.06 (1.02; 1.10)** | **1.07 (1.04; 1.11)** |
| Melanoma | 437,124/1,893 | 1.07 (1.02; 1.12) | 1.06 (1.01; 1.12) | 1.03 (0.98; 1.08) | 1.06 (1.01; 1.12) | 0.98 (0.94; 1.03) | 1.03 (0.98; 1.09) |
| Multiple Myeloma | 437,306/763 | 1.11 (1.03; 1.19) | 1.06 (0.98; 1.14) | 1.02 (0.95; 1.10) | 1.06 (0.98; 1.14) | 1.09 (1.01; 1.18) | 1.10 (1.02; 1.19) |
| Non-Hodgkin | 437,155/1,681 | 1.04 (0.99; 1.10) | 1.02 (0.97; 1.07) | 0.99 (0.94; 1.05) | 1.02 (0.97; 1.07) | 1.04 (0.99; 1.09) | 1.04 (0.98; 1.09) |
| Oesophagus | 241,662/297 | **1.24 (1.11; 1.38)** | **1.22 (1.09; 1.36)** | 1.19 (1.05; 1.35) | **1.22 (1.09; 1.36)** | 1.18 (1.05; 1.32) | **1.27 (1.14; 1.43)** |
| Oral | 241,661/290 | 0.96 (0.85; 1.09) | 0.98 (0.87; 1.12) | 0.90 (0.79; 1.02) | 0.98 (0.87; 1.12) | 0.95 (0.84; 1.08) | 0.97 (0.85; 1.10) |
| Ovary | 235,111/0,852 | 1.01 (0.94; 1.09) | 1.01 (0.94; 1.09) | 1.03 (0.95; 1.11) | 1.01 (0.94; 1.09) | 1.03 (0.96; 1.11) | 1.03 (0.95; 1.10) |
| Pancreas | 437,271/1,136 | **1.13 (1.06; 1.19)** | 1.08 (1.02; 1.15) | **1.11 (1.04; 1.18)** | 1.08 (1.02; 1.15) | 1.09 (1.02; 1.15) | **1.11 (1.04; 1.18)** |
| Stomach | 437,294/0,747 | **1.24 (1.16; 1.32)** | **1.14 (1.06; 1.22)** | **1.17 (1.08; 1.26)** | **1.14 (1.06; 1.22)** | **1.21 (1.13; 1.30)** | **1.23 (1.14; 1.32)** |
| S. Cardia | 437,338/0,404 | **1.35 (1.24; 1.48)** | **1.24 (1.13; 1.36)** | **1.28 (1.15; 1.42)** | **1.24 (1.13; 1.36)** | **1.29 (1.17; 1.42)** | **1.36 (1.23; 1.49)** |
| S. No Cardia | 437,370/0,187 | 1.11 (0.96; 1.27) | 1.01 (0.87; 1.17) | 1.08 (0.93; 1.26) | 1.01 (0.87; 1.17) | **1.26 (1.09; 1.45)** | 1.14 (0.99; 1.32) |
| Prostate | 201,290/7,250 | 0.97 (0.94; 0.99) | 0.96 (0.94; 0.99) | **0.95 (0.92; 0.97)** | 0.96 (0.94; 0.99) | 0.99 (0.96; 1.01) | 0.97 (0.94; 0.99) |
| Testis | 202,114/66 | 0.91 (0.70; 1.18) | 0.91 (0.69; 1.19) | 0.90 (0.69; 1.16) | 0.91 (0.69; 1.19) | 0.93 (0.72; 1.21) | 0.90 (0.68; 1.17) |
| Thyroid | 437,340/284 | 1.12 (1.00; 1.26) | 1.11 (0.99; 1.24) | 1.11 (0.98; 1.26) | 1.11 (0.99; 1.24) | **1.24 (1.10; 1.39)** | **1.22 (1.08; 1.37)** |
| Uterine | 235,061/1,188 | **1.68 (1.61; 1.76)** | **1.59 (1.52; 1.67)** | **1.70 (1.60; 1.82)** | **1.59 (1.52; 1.67)** | **1.26 (1.20; 1.34)** | **1.66 (1.57; 1.75)** |

_Data is presented as Hazar Ratio and their 95% confidence interval. Analyses were adjusted age, sex, ethnicity, education, deprivation, smoking, dietary intake (alcohol, fruits & vegetables, red & processed meat, and oily fish), discretionary sedentary behaviour physical activity and height. BMI: Body Mass Index, BF%: Body Fat Percentage, WHR: waits hip ratio, WHTR: Waist height ratio, HC: hip circumference. P-values are corrected for multiple testing by using the Holm’s method. In_ **_bold_** _are those associations statistically significant after correcting for multiple testing. Participants classified as underweight (BMI < 18.5 kg/m2 were excluded from the analyses (n = 2629)_

**Table S6:** Association of adiposity markers with mortality from 24 cancer sites per 1 SD increase in adiposity markers with height as covariate.

| **Cancer site** | **Total/Event** | **BMI** | **WC** | **BF%** | **HC** | **WHR** | **WHTR** |
| --- | --- | --- | --- | --- | --- | --- | --- |
| **Overall** | 436,695/11,265 | **1.06 (1.04; 1.08)** | 1.03 (1.01; 1.05) | 1.06 (1.04; 1.08) | **1.11 (1.09; 1.14)** | **1.09 (1.07; 1.11)** | 1.03 (1.01; 1.05) |
| Bladder | 437,383/301 | 1.11 (0.99; 1.24) | 1.10 (0.99; 1.24) | 1.09 (0.97; 1.24) | 1.13 (1.01; 1.27) | 1.16 (1.03; 1.31) | 1.10 (0.99; 1.24) |
| Brain | 437,339/578 | 0.93 (0.85; 1.01) | 0.90 (0.82; 0.98) | 0.98 (0.90; 1.07) | 0.95 (0.87; 1.04) | 0.90 (0.82; 0.98) | 0.90 (0.82; 0.98) |
| Breast | 235,252/477 | 1.13 (1.03; 1.24) | 1.11 (1.01; 1.21) | 1.17 (1.05; 1.29) | 1.10 (1.00; 1.21) | 1.15 (1.04; 1.26) | 1.11 (1.01; 1.21) |
| Breast Postmenopausal | 140,365/307 | 1.16 (1.03; 1.30) | 1.11 (0.98; 1.24) | 1.18 (1.04; 1.35) | 1.16 (1.03; 1.30) | 1.18 (1.05; 1.33) | 1.11 (0.98; 1.24) |
| Breast Premenopausal | 58,935/89 | 1.18 (0.97; 1.43) | 1.16 (0.95; 1.41) | 1.21 (0.98; 1.50) | 0.99 (0.79; 1.24) | 1.11 (0.89; 1.38) | 1.16 (0.95; 1.41) |
| Cervix | 235,263/21 | 1.24 (0.83; 1.85) | 1.13 (0.74; 1.71) | 1.37 (0.85; 2.20) | 1.00 (0.64; 1.55) | 1.10 (0.70; 1.71) | 1.13 (0.74; 1.71) |
| Colorectal | 437,336/1,151 | **1.11 (1.04; 1.18)** | 1.07 (1.01; 1.14) | 1.10 (1.03; 1.17) | **1.15 (1.08; 1.22)** | **1.15 (1.08; 1.22)** | 1.07 (1.01; 1.14) |
| Colon | 437,355/655 | 1.12 (1.03; 1.21) | 1.10 (1.02; 1.19) | 1.08 (0.99; 1.18) | 1.13 (1.04; 1.22) | **1.16 (1.07; 1.26)** | 1.10 (1.02; 1.19) |
| Distal | 437,392/91 | 0.85 (0.67; 1.07) | 0.95 (0.76; 1.20) | 0.95 (0.76; 1.19) | 0.96 (0.77; 1.20) | 0.94 (0.75; 1.19) | 0.95 (0.76; 1.20) |
| Proximal | 437,390/156 | 1.17 (1.00; 1.37) | 1.11 (0.95; 1.31) | 1.16 (0.97; 1.38) | 1.26 (1.08; 1.48) | 1.26 (1.07; 1.48) | 1.11 (0.95; 1.31) |
| Rectum | 437,374/498 | 1.10 (1.00; 1.21) | 1.04 (0.95; 1.14) | 1.13 (1.02; 1.24) | **1.17 (1.07; 1.28)** | 1.13 (1.03; 1.25) | 1.04 (0.95; 1.14) |
| Endometrium | 235,263/128 | **1.71 (1.48; 1.98)** | **1.56 (1.35; 1.81)** | 1.84 (1.50; 2.26) | **1.35 (1.14; 1.60)** | **1.70 (1.44; 2.01)** | **1.56 (1.35; 1.81)** |
| Gallbladder | 437,389/57 | 1.38 (1.09; 1.75) | 1.31 (1.04; 1.66) | 1.61 (1.20; 2.16) | 1.41 (1.11; 1.79) | **1.51 (1.18; 1.94)** | 1.31 (1.04; 1.66) |
| Kidney | 437,371/327 | **1.32 (1.19; 1.46)** | **1.22 (1.10; 1.35)** | 1.30 (1.15; 1.46) | **1.36 (1.23; 1.50)** | **1.38 (1.24; 1.54)** | **1.22 (1.10; 1.35)** |
| Leukaemia | 437,367/405 | 1.11 (1.00; 1.23) | 1.09 (0.99; 1.21) | 1.06 (0.95; 1.18) | 1.02 (0.92; 1.12) | 1.08 (0.97; 1.20) | 1.09 (0.99; 1.21) |
| Liver | 437,361/434 | **1.32 (1.21; 1.44)** | **1.25 (1.14; 1.36)** | 1.37 (1.23; 1.52) | **1.31 (1.20; 1.44)** | **1.39 (1.27; 1.52)** | **1.25 (1.14; 1.36)** |
| Lung | 241,684/246 | 0.97 (0.84; 1.11) | 0.97 (0.84; 1.12) | 0.91 (0.80; 1.05) | 0.97 (0.84; 1.12) | 1.03 (0.90; 1.17) | 1.00 (0.87; 1.16) |
| Lymphatic | 437,333/1,063 | **1.11 (1.05; 1.18)** | 1.07 (1.01; 1.14) | 1.03 (0.96; 1.10) | 1.08 (1.02; 1.15) | 1.10 (1.03; 1.18) | 1.07 (1.01; 1.14) |
| Melanoma | 437,387/180 | 1.06 (0.90; 1.24) | 1.07 (0.91; 1.25) | 1.02 (0.87; 1.20) | 0.92 (0.79; 1.08) | 0.99 (0.84; 1.17) | 1.07 (0.91; 1.25) |
| Multiple Myeloma | 437,383/221 | 1.15 (1.00; 1.32) | 1.17 (1.02; 1.34) | 1.03 (0.89; 1.19) | 1.00 (0.87; 1.15) | 1.12 (0.97; 1.29) | 1.17 (1.02; 1.34) |
| Non-Hodgkin | 437,372/416 | 1.07 (0.97; 1.19) | 0.98 (0.89; 1.09) | 0.98 (0.88; 1.09) | **1.18 (1.07; 1.30)** | 1.10 (0.99; 1.21) | 0.98 (0.89; 1.09) |
| Oesophagus | 241,696/152 | **1.35 (1.16; 1.56)** | **1.37 (1.19; 1.57)** | **1.30 (1.10; 1.55)** | **1.37 (1.19; 1.57)** | 1.22 (1.04; 1.43) | **1.41 (1.21; 1.65)** |
| Oral | 241,700/45 | 0.86 (0.63; 1.18) | 0.79 (0.57; 1.10) | 0.94 (0.69; 1.28) | 0.79 (0.57; 1.10) | 1.06 (0.78; 1.43) | 0.90 (0.65; 1.23) |
| Ovary | 235,252/390 | 1.02 (0.91; 1.14) | 1.03 (0.92; 1.14) | 1.03 (0.92; 1.15) | 0.96 (0.86; 1.06) | 0.99 (0.89; 1.11) | 1.03 (0.92; 1.14) |
| Pancreas | 437,320/911 | **1.13 (1.06; 1.21)** | 1.09 (1.02; 1.16) | 1.11 (1.03; 1.19) | 1.11 (1.04; 1.19) | **1.13 (1.05; 1.21)** | 1.09 (1.02; 1.16) |
| Prostate | 202,112/632 | 1.04 (0.96; 1.14) | 1.02 (0.93; 1.11) | 1.01 (0.93; 1.10) | 1.11 (1.03; 1.21) | 1.08 (1.00; 1.18) | 1.02 (0.93; 1.11) |
| Stomach | 437,368/318 | **1.22 (1.10; 1.36)** | 1.11 (1.00; 1.24) | 1.09 (0.97; 1.23) | **1.20 (1.07; 1.34)** | **1.21 (1.08; 1.35)** | 1.11 (1.00; 1.24) |
| S. Cardia | 437,390/58 | **1.58 (1.27; 1.95)** | 1.35 (1.09; 1.68) | 1.48 (1.12; 1.97) | **1.55 (1.30; 1.86)** | **1.64 (1.30; 2.08)** | 1.35 (1.09; 1.68) |
| Thyroid | 437,390/19 | 1.05 (0.64; 1.70) | 1.02 (0.63; 1.66) | 1.23 (0.74; 2.03) | 1.38 (0.93; 2.05) | 1.21 (0.75; 1.94) | 1.02 (0.63; 1.66) |
| Uterine | 235,260/193 | **1.55 (1.37; 1.76)** | **1.46 (1.28; 1.65)** | 1.59 (1.35; 1.87) | 1.22 (1.06; 1.41) | **1.51 (1.32; 1.74)** | **1.46 (1.28; 1.65)** |

_Data is presented as Hazar Ratio and their 95% confidence interval. Analyses were adjusted age, sex, ethnicity, education, deprivation, smoking, dietary intake (alcohol, fruits & vegetables, red & processed meat, and oily fish), discretionary sedentary behaviour, physical activity and height. BMI: Body Mass Index, BF%: Body Fat Percentage, WHR: waits hip ratio, WHTR: Waist height ratio, HC: hip circumference. P-values are corrected for multiple testing by using the Holm’s method. In_ **_bold_** _are those associations statistically significant after correcting for multiple testing. Participants classified as underweight (BMI < 18.5 kg/m2 were excluded from the analyses (n = 2629)_
